# *Vibrio cholerae* in Water Environments: A Systematic Review and Meta‐Analysis

**DOI:** 10.1111/1758-2229.70103

**Published:** 2025-05-29

**Authors:** Aaron Awere‐Duodu, Onyansaniba K. Ntim, Eric S. Donkor

**Affiliations:** ^1^ Department of Medical Microbiology University of Ghana Medical School Accra Ghana

**Keywords:** cholera, diarrhea, prevalence, *Vibrio cholerae*, water environments

## Abstract

*Vibrio cholerae* is a water‐borne pathogen transmitted via the faecal‐oral route, with water being a major vehicle for infection. The pathogen has caused seven pandemics in history, with contaminated water identified as the infection source. Seasonal outbreaks, claiming approximately 21,000–143,000 lives yearly, are facilitated by contaminated water environments. This systematic review, therefore, determined the prevalence of *V. cholerae* in water environments. A comprehensive literature search was conducted in PubMed, Web of Science, and SCOPUS. After the screening process, 87 articles were included in our study. RStudio version 4.3.3 was used in conducting our meta‐analysis with the data subjected to the random‐effects model. The included articles were from 38 countries, which spanned 6 continents. The prevalences of *V. cholerae* in water environments were as follows: drinking water (15.69%), untreated sewage (57.26%), treated sewage (95.18%), surface water (41.95%), groundwater (26.23%), and others (20.81%). Continental prevalence revealed the following: Australia (85.00%), North America (66.60%), Africa (42.07), South America (39.32%), Asia (29.28%), and Europe (24.48%). There is a high prevalence of *V. cholerae* in water environments. More effective water treatment methods are needed to drastically reduce its prevalence to insignificant levels, especially in treated drinking water.

## Graphical abstract

Water facilitates the fecal‐oral transmission of cholera due to the interconnectedness of all water environments. Understanding the current levels of *Vibrio cholerae* contamination in these water environments is crucial to tackling the ongoing cholera pandemic. Therefore, this systematic review and meta‐analysis determined the global prevalence of *V. cholerae* in water environments.

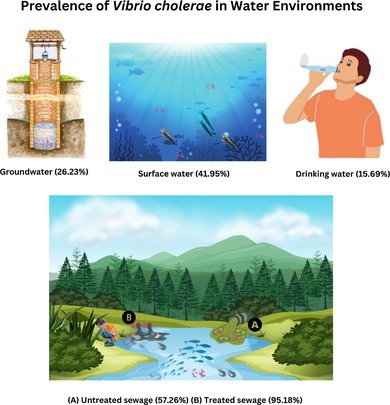

## 1 Introduction

*Vibrio cholerae* is the pathogenic agent of one of the oldest infectious diseases known to humans–cholera (Deen et al. 2020). It has caused seven pandemics, with the first six occurring from 1817 to 1923 and the seventh ongoing in 47 countries since it began in 1961 (Piret and Boivin 2021; Bhandari et al. 2021). The seventh pandemic has resulted in major epidemics in many regions of the world, with notable outbreaks in Haiti, Ghana, Mexico, and Yemen in recent times (Piret and Boivin 2021). The World Health Organization (WHO) recently reported that in 2023, 535,321 cases of cholera occurred in 45 countries, resulting in 4007 deaths (WHO 2024). Researchers have estimated that there are approximately 1.3–4 million cases of cholera, resulting in approximately 21,000–143,000 deaths globally each year (WHO 2024; Strip 2024). However, these numbers may represent only a fraction of the true burden of cholera due to underreporting (Piret and Boivin 2021). This underestimation could explain why no official estimates of morbidity and mortality rates exist for all cholera pandemics to date.

There are four serogroups of *V. cholerae* : O1, O139, non‐O1, and non‐O139 serogroups, which are characterised according to their lipopolysaccharide O antigens (Pal et al. 2023). The O1 and O139 serogroups are known as toxigenic *V. cholerae* , with the O1 serogroup made up of two strains, namely the classical and El Tor strains (Bhandari et al. 2021). The former caused the first six pandemics, and the latter, the current pandemic (De 2021). The El Tor strain is reported to have evolved from the classical strain, with reports of co‐detections in the early stages of the pandemic (Piret and Boivin 2021). The O1 and O139 serogroups, responsible for the cholera pandemics and outbreaks, respectively, produce the cholera toxin (CT) and the toxin co‐regulated pilus (TCP), which facilitate intestinal colonization and disease progression (WHO 2024; Bhandari et al. 2023). Conversely, the majority of the non‐O1 and non‐O139 serogroups do not produce the CT and TCP virulence factors, but rather other virulent facilitators, and have been found to cause invasive and extra‐intestinal infections (Ramamurthy et al. 2020).

*V. cholerae* is a water‐borne pathogen with an infectious dose of 10^2^–10^6^ infectious colony‐forming units that is transmitted via the fecal‐oral route, making contaminated drinking water and food major infection vehicles (Bhandari et al. 2023). Unlike other notable pathogenic *Vibrio* species, such as *Vibrio parahaemolyticus* and *Vibrio vulnificus* , that require high salinity environments like marine waters for survival, *V. cholerae* can persist in marine waters as well as brackish and freshwater environments due to its favorably low 15% salinity requirement for optimum growth in water environments (Piret and Boivin 2021; Kokashvili et al. 2015). These factors make it a natural inhabitant of major water environments and a significant threat to public health.

The importance of water in all aspects of human lives, be it domestic, agricultural, or industrial purposes, highlights the need to ensure the production and use of clean and safe water to maintain a healthy population. This makes water an indispensable factor in the WHO's target to end the ongoing cholera pandemic by 2030 (WHO 2024). Despite water being a major transmission vehicle for *V. cholerae* , to the best of our knowledge, no systematic review has been conducted to determine its global prevalence in water environments. Therefore, this systematic review and meta‐analysis provides the first comprehensive synthesis of the global prevalence of *V. cholerae* in water environments, as well as its antibiotic resistance patterns. We aim to inform treatment practices, as well as prevention and transmission policies with our findings.

## 2 Methodology

### 2.1 Search Strategy

This study employed the Preferred Reporting Items for Systematic Reviews and Meta‐Analyses (PRISMA) guidelines of 2020 (Page et al. 2021). The study protocol was registered in Open Science Frame (OSF) and available at https://doi.org/10.17605/OSF.IO/35CHS. A search string was designed from the keywords “ *Vibrio cholerae* ” and “water”. Relevant synonyms were combined in a phrase using the Boolean words “OR” and “AND”. The search results were limited to studies conducted from 2000 to 2024. The search string used was as follows:

(“ *Vibrio cholerae* ” OR “ *V. cholerae* ” OR “Cholera”) AND (“X”).

The specific water environments denoted as “X” are presented in Table 1.

**TABLE 1:** Search strategy.

Type of water environment	Boolean search phrase
Drinking water	“Drinking water” OR “Tap water” OR “Potable water”
Untreated sewage	“Untreated waste water” OR “Untreated sewage” OR “Untreated wastewater”
Treated sewage	“Treated waste water” OR “Treated sewage” OR “Treated wastewater”
Surface water	“Stream” OR “River” OR “Lake” OR “Freshwater” OR “Seawater” OR “Marine water”
Groundwater	“Well water” OR “Spring water” OR “Groundwater”

### 2.2 Study Selection

Two independent authors performed the screening process, with the third author serving as an arbiter in instances of discrepancies. The criteria used for the study selection are as follows:

Inclusion criteria:

1. Quantitative primary study;
2. Studies in English; and,
3. Contained prevalence data of *V. cholerae* in a defined water environment.

Exclusion criteria:

1. Qualitative studies;
2. Systematic reviews, meta‐analyses, narrative reviews, case series, and case reports;
3. Less than or equal to (≤)10 samples;
4. No *V. cholerae* detected; and,
5. No prevalence data on *V. cholerae* .

### 2.3 Data Extraction

Data for the following relevant variables in the included studies were extracted: first author's last name, country in which the study was conducted, study period, water environments sampled, bacteria concentration method, bacteria identification method, bacteria DNA extraction method, molecular detection method, *V. cholerae* virulence genes detected, *V. cholerae* serogroups, and antibiotic resistance. Prevalence data for *V. cholerae* in defined water environments, comprising positive water samples and total water samples tested, were extracted for meta‐analysis.

### 2.4 Quality Assessment

The quality of the included articles was determined using assessment tools for prevalence studies modified from Hoy et al. (Hoy et al. 2012). The tools comprised external and internal assessment variables. The external variables included target population representation, adequate number of study samples, random sampling, and sample size calculation. The internal validity tools were the definition of the water environment, identification method, data collection methods, duration of study, and reporting of prevalence calculation parameters. Each of the included studies was subjected to these tools to determine study quality (risk of bias) as low risk (score = 7–9), moderate risk (score = 4–6), and high risk (score = 0–3) (Supplementary Table 5).

### 2.5 Data Analysis

Our meta‐analysis was conducted with RStudio version 4.3.3, using the meta package. The Freeman–Tukey arcsine transformation was used to stabilize variances across included articles, facilitating pooled prevalence generation using the DerSimonian–Laird method. The confidence intervals of individual articles were determined by the Clopper–Pearson method. The I^2^ statistic with values 25%, 50%, and ≥ 75% indicating low, moderate, and high was used in assessing heterogeneity. The confidence intervals of the *I* ^2^ statistic were determined by the Jackson method. Publication bias was visually and statistically assessed using the funnel plot and Egger's regression test, respectively. Meta‐regression and sensitivity analysis were performed to determine the sources of heterogeneity and robustness of the included articles, respectively. Statistical significance was set at a *p*‐value of < 0.05.

## 3 Results

### 3.1 Search Results

**FIGURE 1:**
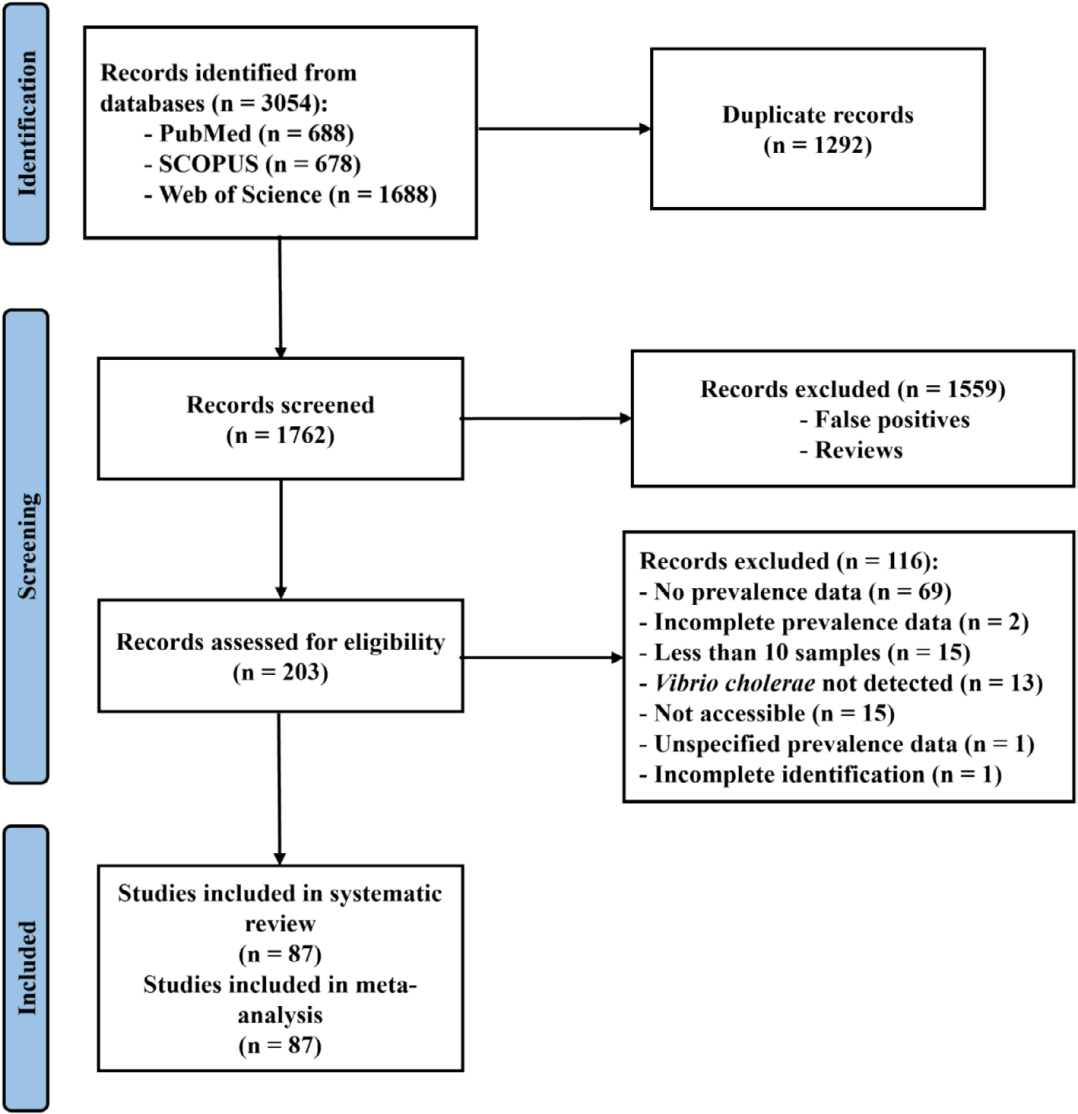
PRISMA flowchart illustrating the screening process.

A thorough search was conducted in August 2024 in PubMed, SCOPUS, and Web of Science to retrieve relevant articles for this study. In total, 3054 articles were retrieved from our search, comprising 2023 duplicates. After resolving the duplicates, 1292 were deleted, leaving us with 1762 articles to be screened. The screening process was done chronologically as follows: title screening, abstract screening, and full‐text screening. Two hundred and three (203) eligible articles underwent full‐text screening, with 116 articles excluded for specific reasons (Supplementary Table 4), and 87 articles (Bhandari et al. 2023; Kokashvili et al. 2015; Abana et al. 2019; Ahmad et al. 2014; Ahmed et al. 2020; Akoachere and Mbuntcha 2014; Alam et al. 2015; Alaoui et al. 2010; Aulet et al. 2007; Bahk et al. 2020; Bauza et al. 2020; Bisimwa et al. 2022; Bliem et al. 2018; Böer et al. 2013; Bwire et al. 2018; Chandran et al. 2008; Chaturongkasumrit et al. 2013; Chigbu and Iroegbu 2000; Chomvarin et al. 2007; Coly et al. 2013; Dickinson et al. 2013; du Preez et al. 2010a; Dumontet et al. 2000; El‐Sayed et al. 2019; Fang et al. 2019; Faouzi et al. 2023; Ferdous et al. 2018; Ferguson et al. 2012; Fernández‐Delgado et al. 2017; Fraga et al. 2007; George et al. 2018; Gil et al. 2004; Goh et al. 2017; Grothen et al. 2017; Halder et al. 2017; Hosen et al. 2021; Hounmanou et al. 2019; Islam et al. 2011; Jesudason et al. 2000; Kaboré et al. 2018; Kachienga et al. 2024; Kaddumukasa et al. 2012; Kahler et al. 2015; Keawvichit et al. 2001; Kim et al. 2023; Kirschner et al. 2018; Lee et al. 2019; Lipp et al. 2003; Luo et al. 2021; Malayil et al. 2011; Mathews et al. 2018; Mishra et al. 2011; Mogessie et al. 2024; Mok et al. 2019; Momtaz et al. 2013; Mookerjee et al. 2014, 2015; Nayak et al. 2020; Ng et al. 2018; Onyuka et al. 2011; Pal et al. 2021; Palit et al. 2012; Potgieter et al. 2020; Rafique et al. 2016; Rai et al. 2019; Rasheed et al. 2009; Sacheli et al. 2023; Saima et al. 2023; Saravanan et al. 2007; Schriewer et al. 2010; Shanan et al. 2011; Shishir et al. 2018; Singh and Lin 2015; Sirajul Islam et al. 2007; Sorensen et al. 2015; Ssemanda et al. 2018; Taviani et al. 2022; Teklehaimanot et al. 2015; Thongchankaew et al. 2011; Torresi et al. 2018; Wang et al. 2020; Waturangi et al. 2012; Whitehouse et al. 2010; Wongworapat et al. 2001; Wu et al. 2019; Yan et al. 2019; Yue et al. 2014) included in the study (Figure 1).

### 3.2 Description of Study Characteristics

Eighty‐seven articles (87) from six continents were included in this study. Most of these articles were from Asia (10 countries; 42 articles), followed by Africa (17 countries; 24 articles), South America (4 countries; 9 articles), Europe (4 countries; 6 articles), North America (2 countries; 5 articles), and Australia (1 country; 1 article). The articles were conducted from 1996 to 2023 and published from 2000 to 2024. The most used water concentration method was membrane filtration (48 articles). The frequently used bacterial identification methods were a combination of culture/biochemical tests/serological tests and RT‐qPCR (40 articles), culture/biochemical test/serological test (27 articles), and RT‐qPCR (14 articles). The reported *V. cholerae* serogroups were O1, O139, non‐O1, non‐O139, and O22. The most frequently detected virulence genes were ctxA (20 articles), ompW (14 articles), toxR (13 articles), tcpA (11 articles), ctxB (6 articles), and hlyA (6 articles). The reported antibiotic resistance was as follows: Amikacin (11%), Amoxicillin (28–100%), Amoxicillin‐clavulanic acid (14%), Ampicillin (21.3–100%), Augmentin (11–18.8%), Azithromycin (33.33–42.6%), Cefazolin (68.70%), Ceftazidime (82.1%), Ceftriaxone (51.3%), Cefoxitin (9.57–53.8%), Cephalexin (83.33%), Cephalothin (9–60%), Cephamandole (23%), Chloramphenicol (3–8.33%), Ciprofloxacin (9.57–100%), Cotrimoxazole (34.4–100%), Doxycycline (9.4–14.1%), Erythromycin (18–100%), Gentamicin (1.7–100%), Imipenem (27.83%), Kanamycin (3–12%), Meropenem (25.6%), Nalidixic acid (72.5–100%), Nitrofurantoin (3.9–7.3%), Penicillin (100%), Piperacillin/Tazobactam (5.1%), Polymyxin B (12%), Streptomycin (11–62%), Sulfamethazole (8.33%), Tetracycline (1.4–100%), Trimethoprim/Sulphamethoxazole (0.6–69.2%), and Vancomycin (100%) (Supplementary Table 1). The majority of included articles were of moderate risk (82.8%) with the remaining 17.2% being of low risk.

One hundred and forty‐six (146) prevalence data were extracted from the 87 included articles for the meta‐analysis. This study's main categories of water environments were drinking water (21 articles; 27 prevalence data), untreated sewage (7 articles; 12 prevalence data), treated sewage (1 article; 4 prevalence data), surface water (61 articles; 78 prevalence data), and groundwater (8 articles; 8 prevalence data). Defined water environments that could not be classified under any main categories were designated as “others” (15 articles; 17 prevalence data). The extracted prevalence data were grouped into continents: Africa (24 articles; 49 prevalence data), Asia (42 articles; 71 prevalence data), Australia (1 article; 1 prevalence data), Europe (6 articles; 7 prevalence data), North America (5 articles; 9 prevalence data), and South America (9 articles; 9 prevalence data). The prevalence data was also grouped into the World Bank classification of countries: low‐income economies (9 articles; 18 prevalence data), lower‐middle‐income economies (37 articles; 67 prevalence data), upper‐middle‐income economies (24 articles; 41 prevalence data), high‐income economies (16 articles; 19 prevalence data), and unclassified group (1 article; 1 prevalence data).

### 3.3 Prevalence of *Vibrio cholerae* in Water Environments

**FIGURE 2:**
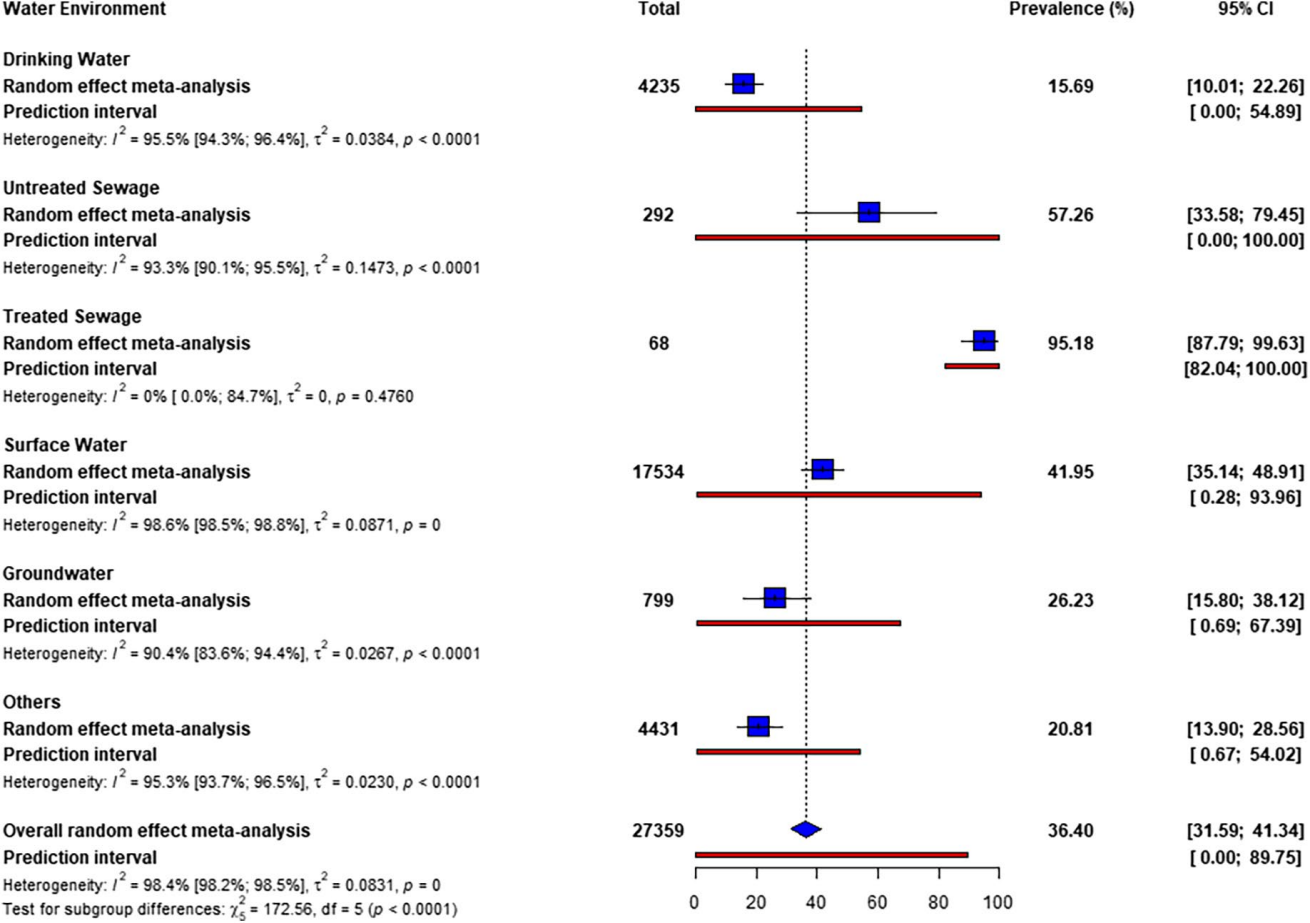
Prevalence of *Vibrio cholerae* in water environments.

The pooled prevalence of *V. cholerae* in the various water environments was 36.40%. The prevalence in the individual water environments was highest in treated sewage (95.18%), followed by untreated sewage (57.26%), and surface water (41.95%), while the lowest prevalence was recorded in drinking water (15.69%) (Figure 2).

### 3.4 Prevalence of *Vibrio cholerae* Serogroups in Water Environments

**FIGURE 3:**
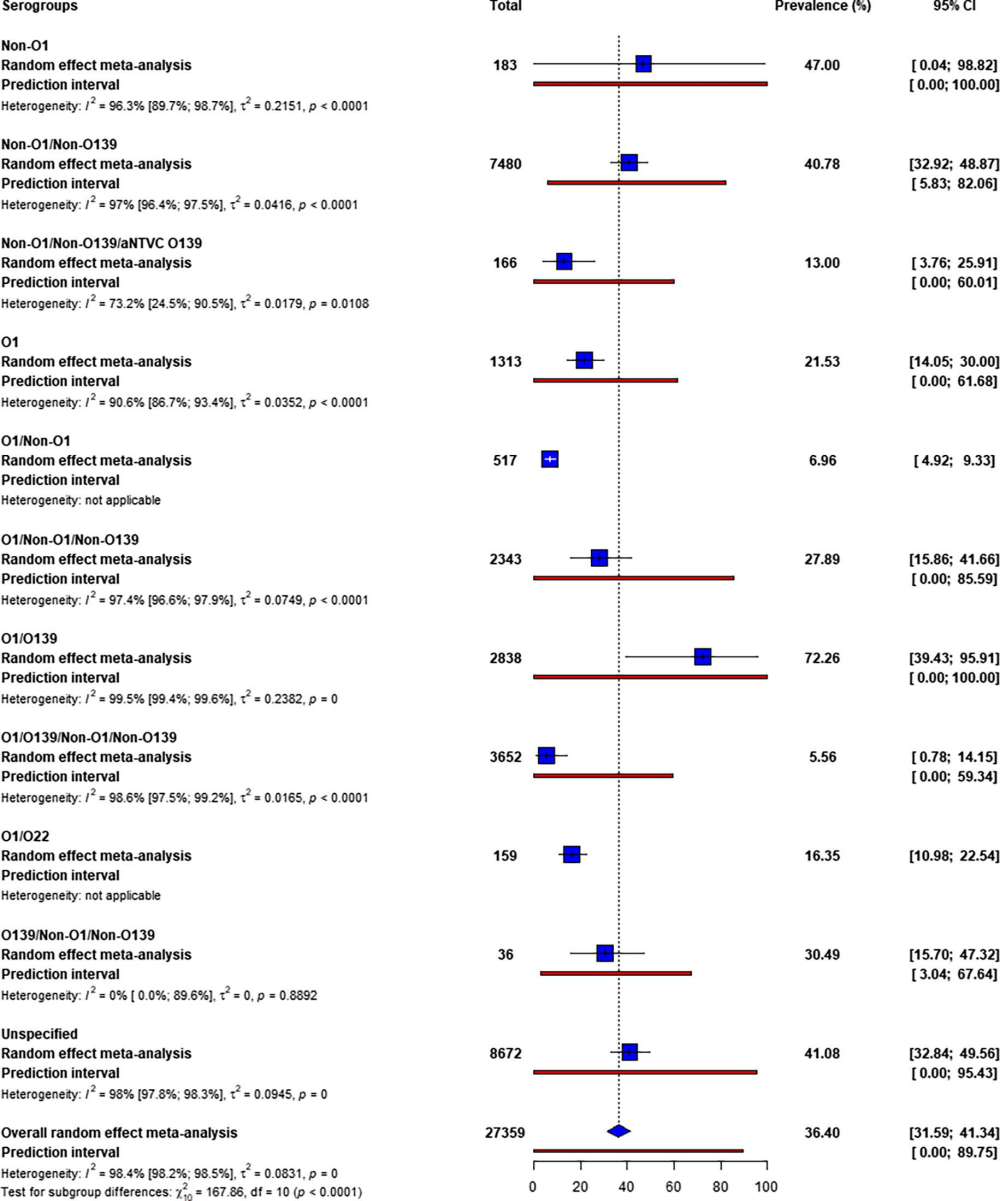
Prevalence of *Vibrio cholerae* serogroups in water environments.

The most prevalent *V. cholerae* serogroups in water environments were O1/O139 (72.26%), followed by non‐O1 (47%), non‐O1/non‐O139 (40.78%), and O139/non‐O1/non‐O139 (30.49%). The least prevalent serogroups detected were O1/non‐O1 (6.96%) and O1/O139/non‐O1/non‐O139 (5.56%) (Figure 3).

### 3.5 Continental and Economic Stratification of *Vibrio cholerae* Prevalence

Continental subgroup analysis showed that Australia (85.00%) had the highest prevalence of *V. cholerae* , followed by North America (66.60%), Africa (42.07%), and South America (39.32%), while Europe (24.48%) had the lowest prevalence (Supplementary Figures 2 and 4).

**FIGURE 4:**
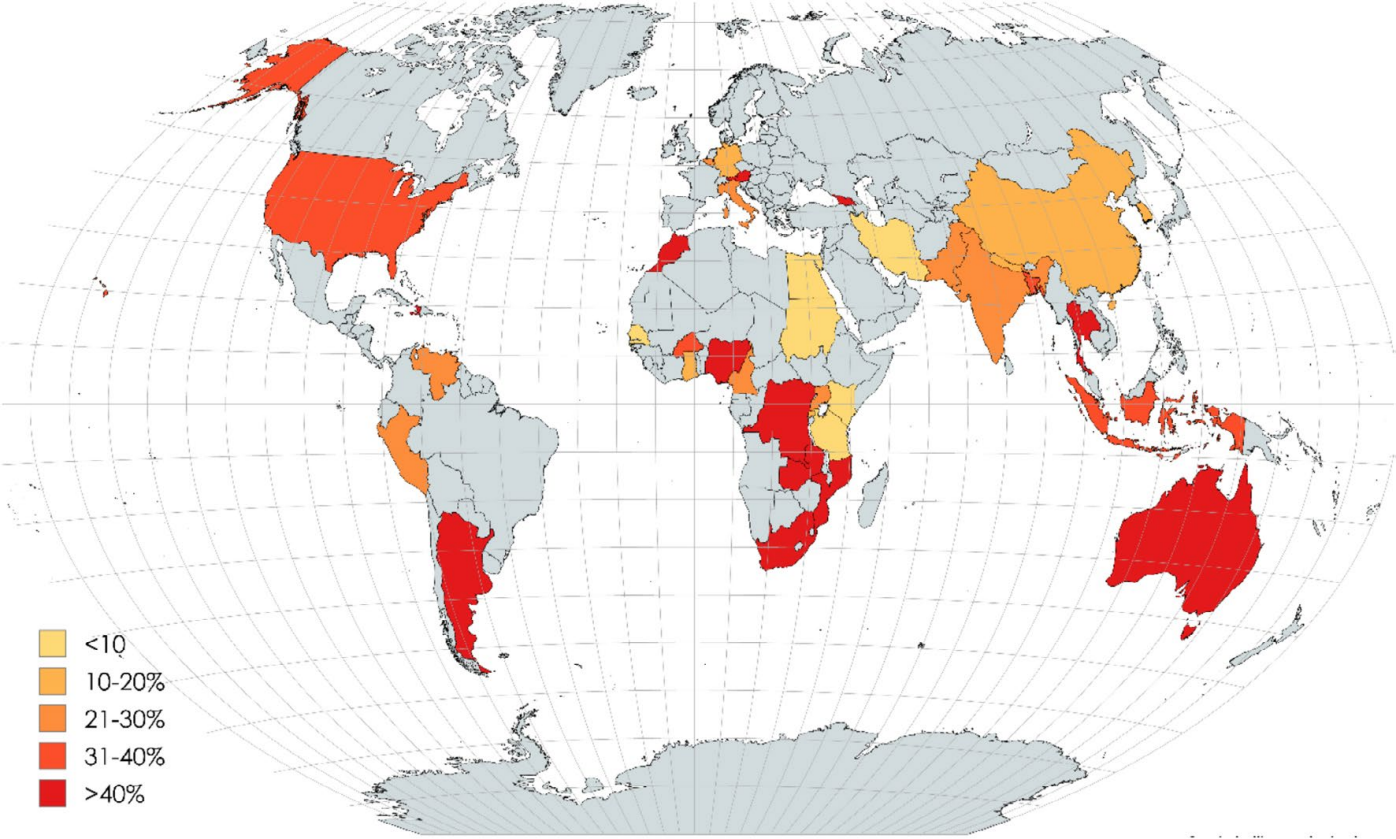
Global repartition of *Vibrio cholerae* prevalence in various water environments.

Economic stratification of *V. cholerae* prevalence showed that upper‐middle‐income economies had the highest prevalence, with 53.66%, followed by lower‐middle‐income economies, with 32.32%, and low‐income economies, with 27.20%. The unclassified group had the lowest prevalence, at 21.74% (Supplementary Figure 3).

### 3.6 Heterogeneity and Publication Bias

The estimated prevalences and pooled prevalence showed significant heterogeneity among the defined water environments. The funnel plot (Supplementary Figure 1) was used to visually assess publication bias in our included articles, which was statistically confirmed with Egger's regression test (*p* = 0.0186).

### 3.7 Subgroup Analysis

The sampling periods for the included articles were grouped into 2000–2011 and 2012–2023 to determine the prevalence of *V. cholerae* in the defined water environments over those periods. Our analysis showed that the prevalence of *V. cholerae* showed a slight decrease from 15.19% [10.01; 22.26] to 14.25% [10.10; 18.93] for the 2000–2011 and 2012–2023 periods, respectively. In untreated sewage, there was a decrease from 40.64% [30.44; 51.23] to 19.92% [13.07; 27.66] for the 2000–2011 and 2012–2023 periods, respectively, with a similar trend observed in groundwater and the others group (Table 2).

**TABLE 2:** Summary of global meta‐analysis results for the prevalence of *Vibrio cholerae* in water environments stratified by sampling period.

Water environment	Prevalence (%) [95% CI]	95% Prediction interval	Number of studies	Number of samples	*H* [95% CI]	*I* ^2^ [95% CI]	*P* heterogeneity
*Drinking water*							
Overall	15.69 [10.01; 22.26]	[0.00; 54.89]	27	4235	4.70 [4.19; 5.26]	95.5 [94.3; 96.4]	**< 0.0001**
2000–2011	15.19 [7.05; 25.42]	[0.00; 54.38]	9	1156	3.55 [2.79; 4.52]	92.1 [87.2; 95.1]	**< 0.0001**
2012–2023	14.25 [10.10; 18.93]	[3.43; 30.14]	10	2089	1.95 [1.42; 2.67]	73.6 [50.4; 86.0]	**< 0.0001**
*Untreated sewage*							
Overall	57.26 [33.58; 79.45]	[0.00; 100.00]	12	292	3.87 [3.19; 4.71]	93.3 [90.1; 95.5]	**< 0.0001**
2000–2011	40.64 [30.44; 51.23]	[24.45; 57.83]	4	93	1.00 [1.00; 2.56]	0.0 [0.0; 84.7]	**0.4461**
2012–2023	19.92 [13.07; 27.66]	[9.34; 32.83]	4	131	1.00 [1.00; 2.56]	0.0 [0.0; 84.7]	0.9960
*Treated sewage*							
Overall	95.18 [87.79; 99.63]	[82.04; 100.00]	4	68	1.00 [1.00; 2.56]	0.0 [0.0; 84.7]	0.4760
2000–2011	—	—	—	—	—	—	—
2012–2023	—	—	—	—	—	—	—
*Surface water*							
Overall	41.95 [35.14; 48.91]	[0.28; 93.96]	78	17,534	8.58 [8.20; 8.97]	98.6 [98.5; 98.8]	**0**
2000–2011	43.07 [31.44; 55.09]	[0.00; 98.54]	33	5304	8.03 [7.47; 8.64]	98.5 [98.2; 98.7]	**0**
2012–2023	45.08 [33.81; 56.60]	[0.51; 96.37]	28	11,143	10.84 [10.17; 11.57]	99.1 [99.0; 99.3]	**0**
*Groundwater*							
Overall	26.23 [15.80; 38.12]	[0.69; 67.39]	8	799	3.23 [2.47; 4.24]	90.4 [83.6; 94.4]	**< 0.0001**
2000–2011	36.29 [10.79; 66.56]	[0.00; 100.00]	3	307	3.87 [2.49; 6.01]	93.3 [83.8; 97.2]	**< 0.0001**
2012–2023	18.60 [7.96; 32.21]	[0.00; 68.99]	4	470	3.02 [1.99; 4.60]	89.1 [74.7; 95.3]	**< 0.0001**
*Others*							
Overall	20.81 [13.90; 28.56]	[0.67; 54.02]	17	4431	4.59 [3.97; 5.32]	95.3 [93.7; 96.5]	**< 0.0001**
2000–2011	35.93 [11.05; 65.21]	[0.00; 100.00]	6	349	4.11 [3.12; 5.42]	94.1 [89.7; 96.6]	**< 0.0001**
2012–2023	20.04 [13.04; 27.98]	[3.76; 43.29]	7	2691	2.87 [2.10; 3.92]	87.8 [77.3; 93.5]	**< 0.0001**

*Note:* 95% CI, 95% confidence interval; *H* is a measure of the extent of heterogeneity; a value of *H* > 1 indicates a potential heterogeneity of the prevalence of rotavirus; *I* ^2^ describes the proportion of total variation in prevalence of rotavirus that is due to heterogeneity; a value > 50% indicates the presence of heterogeneity. Bold indicates significant values.

### 3.8 Meta‐Regression

Sources of heterogeneity in our meta‐analysis were determined by conducting a meta‐regression analysis. The results showed that there were categories in our three major covariates that caused the observed heterogeneity in our meta‐analysis. For the water environments, untreated sewage, treated sewage, and surface water had statistically significant positive effects on the heterogeneity. Regarding continents, Australia and North America had statistically significant positive effects on the heterogeneity. Lower‐middle‐income economies and upper‐middle‐income economies also had statistically significant positive effects on the heterogeneity. Results for Africa and high‐income economies were not generated due to statistical considerations such as collinearity (Supplementary Table 2).

### 3.9 Sensitivity Analysis

The robustness of our included studies was assessed by performing a leave‐one‐out sensitivity analysis. The results indicated that our study selection was robust, with point estimates consistently around 36% and narrow confidence intervals, confirming a high level of confidence for the predicted point estimates (Supplementary Table 3).

## 4 Discussion

Cholera, despite being one of the oldest infectious and most fatal diseases known to humans, continues to pose a significant public health threat, with its most recent pandemic ongoing for more than six decades (Deen et al. 2020). Efforts to ensure the availability of clean and safe drinking water for all have somehow been ineffective, owing to the high levels of pathogenic microorganisms that have been detected in water environments. These challenges are further exacerbated by the current climate change impacts, resulting in a shortage of water in many regions of the world (Gosling and Arnell 2016). While the prevalence of other infectious diarrheal pathogens, such as rotavirus (Awere‐Duodu and Donkor 2024), has been determined in water environments across the world in recent times, that of *V. cholerae* has not been determined. This systematic review and meta‐analysis, therefore, fills this critical gap by presenting the world's first comprehensive synthesis of *V. cholerae* prevalence in water environments. Furthermore, this study provides important information on the antibiotic resistance of the pathogen to inform treatment regimens and to guide effective control policies.

With contaminated water being the cause of all seven cholera pandemics, it was expected that more stringent measures would have been put in place to address the persistence of its pathogen in water environments. Sadly, this was not the case as our meta‐analysis revealed that the pooled prevalence of *V. cholerae* in water environments was 36.40%, with that of drinking water being 15.69%. This prevalence in drinking water is particularly worrying, as contaminated drinking water serves as primary vehicle for cholera transmission (Sirajul Islam et al. 2007). It questions further the effectiveness of the current water treatment systems, as highlighted by a systematic review on rotavirus in water environments (Awere‐Duodu and Donkor 2024), another fatal diarrheal pathogen.

The prevalence of *V. cholerae* in untreated sewage was 57.26%. This was an expected finding due to human excreta being a common habitat for intestinal pathogens, including *V. cholerae* . Surprisingly, the prevalence of *V. cholerae* in treated sewage water was very high at 95.18%. This result is, however, unreliable due to its prevalence data coming from only one study (Teklehaimanot et al. 2015), making it unrepresentative of the true prevalence of *V. cholerae* in treated sewage. Hence, we recommend that more studies on the prevalence of *V. cholerae* in treated sewage should be conducted globally to help ascertain the effectiveness of sewage treatment systems against *V. cholerae*.

Surface water and groundwater had prevalences of 41.95% and 26.23%, respectively. The relatively high prevalence in groundwater may be attributed to the increased use of untreated and treated sewage water for irrigation, a practice necessitated by water shortages caused by climate change. This practice facilitates the infiltration of pathogenic organisms, including *V. cholerae* , into groundwater, highlighting the urgent need to use safer water sources for irrigation. The prevalence of *V. cholerae* in the “others” category of the water environments was 20.81%, likely reflecting its high prevalence in primary water sources such as surface water and groundwater. The high prevalence of *V. cholerae* in natural water sources in this study could also be attributed to the occurrence of natural hosts of the pathogen, such as fish and shrimp, in these water environments (Halpern and Izhaki 2017; Chen et al. 2022).

The toxigenic serogroups O1/O139 (72.26%) were the most prevalent in the water environments. This finding contrasts with the reports of uncommon detection of toxigenic *V. cholerae* serogroups in natural water environments outside outbreak seasons (Bwire et al. 2018; Du Preez et al. 2010b; Alam et al. 2014). Most studies investigating the presence of *V. cholerae* in water environments are usually conducted during outbreak seasons, primarily focusing on these serogroups. The resulting sampling bias likely explains the high prevalence we observed, which could be attributed to fecal contamination of natural water environments during outbreak seasons (Alam et al. 2014). This high prevalence could explain the persistence of the current *V. cholerae* pandemic, owing to the indispensable need for water in several human activities directly affecting our health.

*V. cholerae* , unlike other pathogens, is a major threat to public health in developed and underdeveloped countries alike. Our meta‐analysis revealed irregular patterns of pathogenic prevalence, where poorer countries usually had higher prevalences of disease pathogens. The countries with the highest prevalence of *V. cholerae* were South Africa (93.02%), Haiti (89.66%), Australia (85.00%), Morocco (77.27%), Mozambique (59.16%), the Democratic Republic of Congo (57.99%), Austria (53.99%), and Argentina (52.74%). Stratification of the *V. cholerae* prevalence by continents revealed that Australia (85.00%) had the highest prevalence, followed by North America (66.60%), Africa (42.07%), South America (39.32%), Asia (29.28%), and Europe (24.48%). However, the high prevalence of *V. cholerae* observed in Australia is unreliable due to it being reported by a single study (Bhandari et al. 2023). Similarly, economic stratification according to the World Bank Classification of 2024 revealed that upper‐middle‐income economies (53.66%) had the highest prevalence of *V. cholerae* , followed by lower‐middle‐income economies (32.32%), low‐income economies (27.20%), high‐income economies (26.49%), and the unclassified group (21.74%). The marginal difference in the prevalence of *V. cholerae* in low‐income and high‐income economies is contrary to reports of cholera being a major public health concern in low‐ and middle‐income countries (LMICs) where sanitation conditions are often poor (Chowdhury et al. 2022; Asantewaa et al. 2024).

Our subgroup analysis of the prevalence of *V. cholerae* in water environments over two time periods, 2000–2011 and 2012–2023, revealed notable trends. The prevalence of *V. cholerae* in drinking water showed only a slight decrease, highlighting the pathogen's persistent presence in drinking water across both periods. Interestingly, a significant decrease was observed in the prevalence of *V. cholerae* in untreated sewage, which dropped from 40.64% in 2000–2011 to 19.92% in 2012–2023. This decline may indicate a potential reduction in cholera infections. However, the slight increase in *V. cholerae* prevalence in surface water, from 43.07% to 45.08%, suggests ongoing challenges with sanitation practices, particularly the disposal of untreated sewage into surface water bodies. Additionally, considerable decreases were observed in the prevalence of *V. cholerae* in groundwater and the “others” water sources category, as shown in Table 2.

There may be a higher prevalence of *V. cholerae* in our water environments than reported in this study. *V. cholerae* can be found in water environments as viable and culturable or viable but non‐culturable (Bhandari et al. 2023; Binsztein et al. 2004). The latter, which is often undetected by routine culture and biochemical tests, can only be detected by the RT‐qPCR method (Bhandari et al. 2023). While most of the articles included in our study employed a combination of culture, biochemical, and serological tests, the latter two were the primary methods used for bacterial identification. As a result, viable but non‐culturable strains may have gone undetected.

Cholera causes severe acute watery diarrhea, which requires urgent medical treatment to prevent fatality. Fluid replacement and the administration of antibiotics, irrespective of age, as recommended by the WHO, are required for effective treatment (Chowdhury et al. 2022). Several studies have reported that antibiotics reduce the severity of cholera symptoms (Sharifi‐Mood and Metanat 2014; Davies et al. 2017). However, the emergence of antibiotic resistance poses a great challenge to treatment options. High resistance was observed in the most frequently prescribed antibiotics for cholera patients, specifically, azithromycin (33.33%–42.6%), ciprofloxacin (9.57–100%), cotrimoxazole (34.4–100%), doxycycline (9.4–14.1%), erythromycin (18–100%), tetracycline (1.4–100%), and trimethoprim‐sulfamethoxazole (0.6–69.2%). Additionally, high resistance was observed in high‐end antibiotics such as polymyxin B (12%), meropenem (25.6%), and imipenem (27.83%). These findings pose a great challenge in the medical treatment of cholera globally. While vaccines offer a better alternative to cholera treatment, with prevention being the most effective strategy, the WHO reports that the availability of the three pre‐approved cholera vaccines (Dukoral, Euvichol‐Plus, and Euvichol‐S) remains limited (WHO 2024). These vaccines require two doses for full protection in adults; however, only one‐dose regimens, which provide short‐term protection, are currently being used.

Our meta‐analysis revealed significant heterogeneity, which we assessed using meta‐regression analysis. We identified that the heterogeneity was primarily associated with untreated sewage, treated sewage, and surface water in the water environments, as well as with studies conducted in Australia and North America. The wide prediction intervals observed further reflected the high heterogeneity among the included studies. Additionally, publication bias was evident in the included articles, confirmed by Egger's regression test (*p*‐value = 0.0186). Most of the studies (82.8%) were assessed to have a moderate risk of bias, while the remaining 17.2% had a low risk. Additional potential sources of bias included variations in climate across sampling locations, differences in sampling frequency, and inconsistencies in detection methods.

## 5 Conclusion

The high global prevalence of *V. cholerae* in water environments underscores the urgent need for stricter sanitation practices worldwide. Sanitation campaigns must be intensified within communities to raise awareness and promote good hygiene practices. Given the acute nature and high fatality rate of cholera infections, it is essential to develop more effective water treatment methods to significantly reduce the prevalence of *V. cholerae* , particularly in treated drinking water, to insignificant levels. Additionally, increasing the production of cholera vaccines is crucial to improving their availability and mitigating the frequency of cholera outbreaks globally. Vaccination should be integrated with cholera surveillance programs to facilitate the strategic immunization of at‐risk populations. Furthermore, greater access to vaccines could also reduce the reliance on antibiotics, thereby slowing the development of antibiotic resistance in the pathogen.

## Author Contributions

Conceptualization, E.S.D. and A.A.‐D.; methodology, A.A.‐D. and E.S.D.; software, A.A.‐D. and O.K.N.; validation, E.S.D., A.A.‐D., and O.K.N.; formal analysis, A.A.‐D.; resources, E.S.D., A.A.‐D., and O.K.N.; data curation, A.A.‐D. and O.K.N.; writing – original draft preparation, A.A.‐D. and O.K.N.; writing – review and editing, E.S.D., A.A.‐D., and O.K.N.; visualization, E.S.D., A.A.‐D., and O.K.N.; supervision, E.S.D.; project administration, E.S.D.; funding acquisition, E.S.D. All authors have read and agreed to the published version of the manuscript.

## Conflicts of Interest

The authors declare no conflicts of interest.

## Supporting information


**Supplementary Figure 1:** Funnel plot for studies on *Vibrio cholerae* .
**Supplementary Figure 2**: Continental prevalence of *Vibrio cholerae* in water environments.
**Supplementary Figure 3**: Economic stratification of the prevalences of *Vibrio cholerae* in water environments.
**Supplementary Table 1**: Characteristics of included articles.
**Supplementary Table 2**: Meta‐regression of factors affecting heterogeneity in the study.
**Supplementary Table 3**: Sensitivity analysis of pooled prevalence.
**Supplementary Table 4**: Main reasons for exclusion of eligible studies.

## Data Availability

The data that support the findings of this study are available from the corresponding author upon reasonable request.
