# Supplementary material for: Vibrio cholerae in Water Environments: A Systematic Review and Meta‐Analysis
Source: Environ Microbiol Rep. 2025 May 29;17(3):e70103. doi: 10.1111/1758-2229.70103 (PMC12120263; doi:10.1111/1758-2229.70103)
Supplement: Supplementary file 1 — Supplementary Figure 1: Funnel plot for studies on Vibrio cholerae . Supplementary Figure 2: Continental prevalence of Vibrio cholerae in water environments. Supplementary Figure 3: Economic stratification of the prevalences of Vibrio cholerae in water environments. Supplementary Table 1: Characteristics of included articles. Supplementary Table 2: Meta‐regression of factors affecting heterogeneity in the study. Supplementary Table 3: Sensitivity analysis of pooled prevalence. Supplementary Table 4: Main reasons for exclusion of eligible studies. [file EMI4-17-e70103-s001.docx]

***Vibrio Cholerae* in Water Environments: A Systematic Review and Meta-Analysis**

Aaron Awere-Duodu^1^, Onyansaniba K. Ntim^1^, Eric S. Donkor^1^*

**^1^**Department of Medical Microbiology, University of Ghana Medical School

*Author to whom correspondence should be addressed; [esampane-donkor@ug.edu.gh](mailto:esampane-donkor@ug.edu.gh)

**Supplementary Materials**

**Table of Contents**

**Supplementary Figure 1**: Funnel Plot for Studies on Vibrio cholerae

**Supplementary Figure 2**: Continental Prevalence of Vibrio cholerae in Water Environments

**Supplementary Figure 3**: Economic Stratification of the Prevalences of Vibrio cholerae in Water Environments

**Supplementary Table 1**: Characteristics of Included Articles

**Supplementary Table 2**: Meta-regression of factors affecting heterogeneity in the study

**Supplementary Table 3**: Sensitivity Analysis of Pooled Prevalence

**Supplementary Table 4**: Main Reasons for Exclusion of Eligible Studies


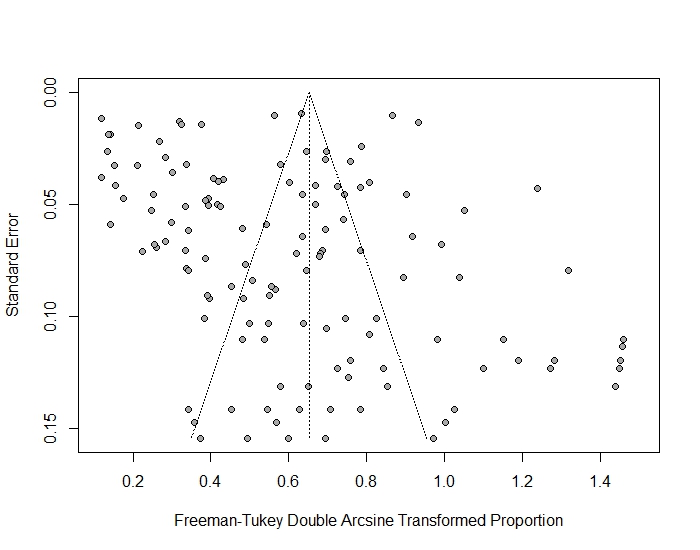
Egger’s regression test p-value = 0.0186

**Supplementary Figure 1**: Funnel Plot for Studies on Vibrio cholerae


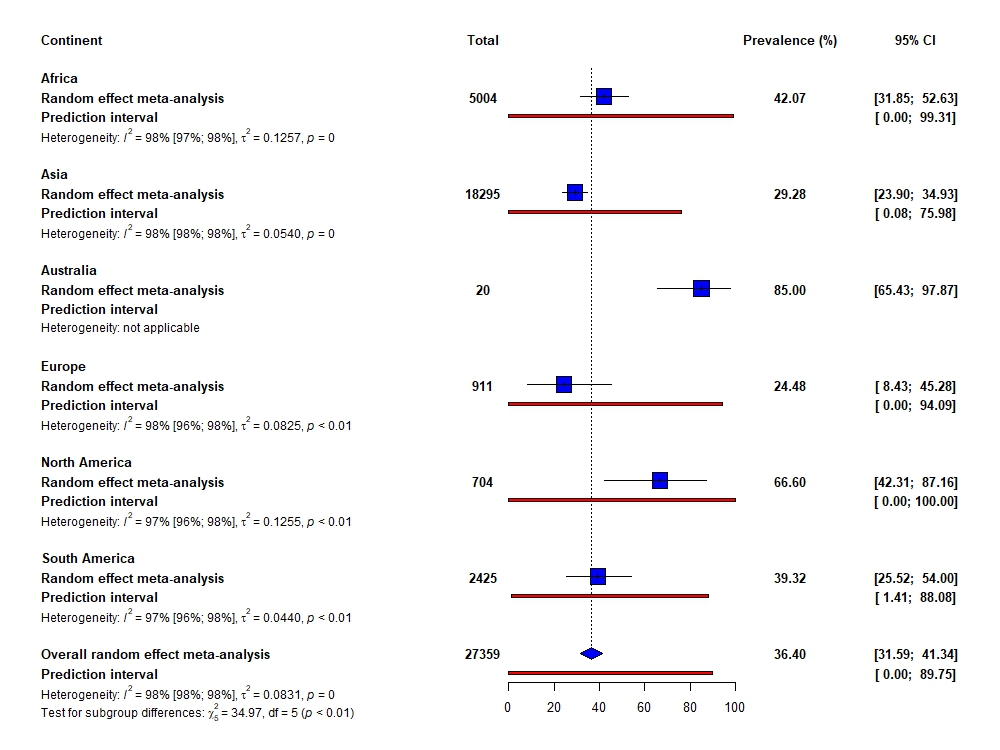


**Supplementary Figure 2**: Continental Prevalence of Vibrio cholerae in Water Environments


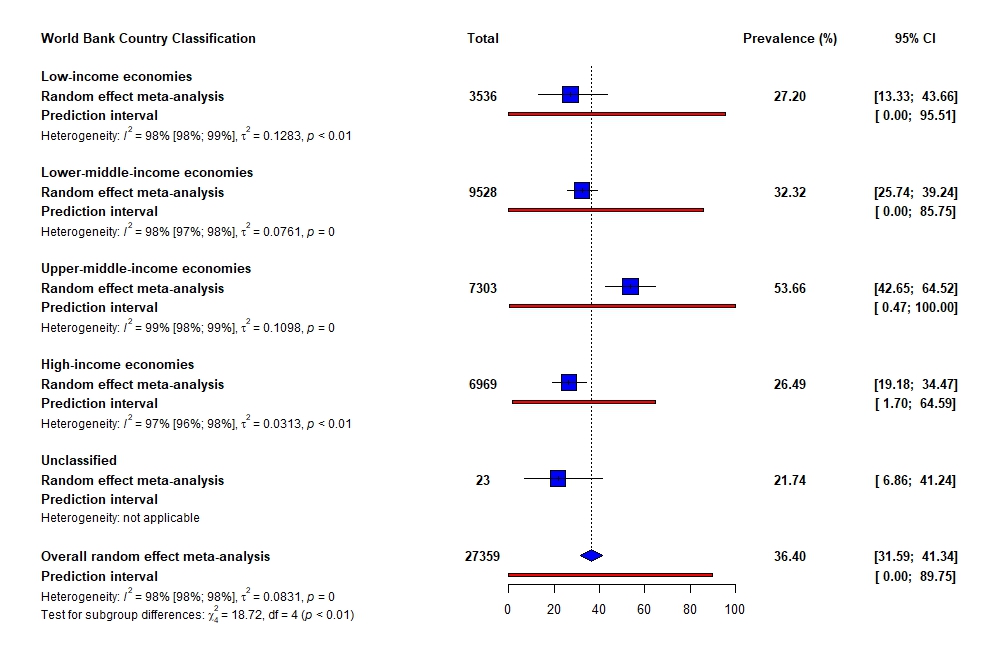


**Supplementary Figure 3**: Economic Stratification of the Prevalences of Vibrio cholerae in Water Environments

**Supplementary Table 1**: Characteristics of Included Articles

| **Publication** | **Country** | **Study period** | **Water environments sampled** | **Bacteria concentration method** | **Bacteria identification method** | **Bacteria DNA extraction method** | **Molecular detection method** | ***Vibrio cholerae* virulence genes detected** | ***Vibrio cholerae* serotypes** | **Antibiotic resistance** |
| --- | --- | --- | --- | --- | --- | --- | --- | --- | --- | --- |
| Abana et al.^1^ | Ghana | October 2015 – January 2016 | Streams, shallow wells, storage tanks, tap water | - | Culture, biochemical analysis, serological test | - | RT-qPCR | Ctx, zot, attRS, ompW, tcpA _El Tor_, tcpA _classical_, rbfO1 | O1 | Erythromycin (92.5%), Nalidixic acid (72.5%) |
| Ahmad et al.^2^ | Pakistan | - | Tube wells, hand pumps, tap water | - | - | - | - | - | - | - |
| Ahmed et al.^3^ | Pakistan | - | Drinking water | Membrane filtration | Culture | - | - | - | - | - |
| Akoachere and Mbuntcha^4^ | Cameroon | February – July 2009 | Wells, tap water, stream | - | Culture, biochemical tests, serological test | - | - | - | O1, non-O1/non-O139 | Amoxicillin (31.3-36%), Augmentin (11-18.8%), Tetracycline (37.5-50%), Doxycycline (9.4-14.1%), Cotrimoxazole (34.4-57.8%), Chloramphenicol (3-6.3%) |
| Alam et al.^5^ | Haiti | Apr 2013 – Mar 2014 | Rivers, estuaries | - | Culture, biochemical tests, serological test | GenElute Bacterial Genomic DNA kit | RT-qPCR, MAMA-PCR | ompW, toxR, tcpAET, ctxA, ctxB, rstRET, rstCET, ctxBCL | O1, non-O1/O139 | Nalidixic acid (100%), Cotrimoxazole (100%) |
| Alaoui et al.^6^ | Morocco | Apr 2004 – Apr 2005 | Wells | Membrane filtration | Culture, biochemical tests, serological test | - | - | - | Non-O1 | Ampicillin (42%), Amoxicillin (28%), amoxicillin-clavulanic acid (14%), cephalothin (60%), cephamandole (23%), cephotaxim (8%), erythromycin (18%), gentamycin (7%), kanamycin (12%), streptomycin (62%), polymyxin B (12%), tetracycline (2 %), sulphamethoxazole (75%), trimethoprim (49%) |
| Aulet et al.^7^ | Argentina | 2003 - 2005 | Rivers | Membrane filtration | Culture, biochemical test, serological test, Direct immunofluorescence of Vibrio cholerae O1 (DFA-DVC) | - | RT-qPCR | ctxA, tcpA | Non-O1/O139 | - |
| Bahk et al.^8^ | South Korea | Aug 2013 – Feb 2019 | Rivers, lakes | Membrane filtration | - | GeneAll Exgene Soil DNA kits | RT-qPCR | - | - | - |
| Bauza et al.^9^ | Kenya | June 2015 | Stored drinking water | Membrane filtration | - | PowerWater DNA Isolation kit | RT-qPCR | ctxA | - | - |
| Bhandari et al.^10^ | Australia | Feb – Mar 2021 | Rivers | Membrane filtration | Culture, MALDI-TOF | - | RT-qPCR | ctxB-Aus, rstR-ET, and tcpA-CC, rstR-CC, tcpA-ET | Non-O1/non-O139 | - |
| Bisimwa et al.^11^ | Democratic Republic of Congo | Jan 2017 – Dec 2019 | Rivers, tributaries | Membrane filtration | Culture | - | - | - | - | - |
| Bliem et al.^12^ | Austria | - | Lakes | Centrifugation | - | MoBio PowerSoil DNA extraction kit | RT-qPCR | ompW | Non-O1/non-139 | - |
| Böer et al.^13^ | Germany | Dec 2009 – Dec 2011 | Coastal waters, sediments | Membrane filtration | Culture, biochemical tests, serological test | Lysozyme/SDS lysis followed by a phenol/chloroform extraction and an isopropanol precipitation | RT-qPCR | - | non-O1/O139 | - |
| Bwire et al.^14^ | Uganda | Feb 2015 – Jan 2016 | Lakes, rivers, ponds, wells | Filtration | Crystal VC dipsticks | Chelex-100 | Multiplex PCR | ompW | non O1/non O139, non-toxigenic atypical V. cholerae O139 | - |
| Chandran et al.^15^ | India | Oct 2004 – Sep 2005 | Lake | - | Culture, biochemical tests | - | - | - | - | - |
| Chaturongkasumrit et al.^16^ | Thailand | 2011 | Flood water, tap water | Membrane filtration, centrifugation | - | NucleoSpin Tissue DNA extraction kit | RT-qPCR | hlyA, ctxA | - | - |
| Chigbu and Iroegbu^17^ | Nigeria | - | Rivers | Membrane filtration | Culture, biochemical tests, microscopy | - | - | - | - | - |
| Chomvarin et al.^18^ | Thailand | 2003 – 2004 | Drinking water, washing water, sewage, river | Filtration | Culture, biochemical tests, serological test | Puregene DNA purification kit | RT-qPCR | ctxA, tcpA | O1, non-O1/non-O139 | - |
| Coly et al.^19^ | Senegal | May 2007 – Dec 2009 | Estuarine water, seawater | Membrane filtration | Culture, biochemical tests, serological test | - | - | - | - | - |
| Dickinson et al.^20^ | United States | May – Sep 2007 | Beach waters | Membrane filtration | - | Cetyltrimethylammonium bromide (CTAB)chloroform-phenol method and a boiling cell lysis method | RT-qPCR | - | - | - |
| du Preez et al.^21^ | Mozambique | Dec 2005 – Feb 2007 | Estuarine water | Membrane filtration | DFA | - | - | - | O1, O139 | - |
| Dumontet et al.^22^ | Italy | May – Sep 1996 | Marine water | - | Culture, serological test | - | - | - | Non-O1 | - |
| El-Sayed et al.^23^ | Egypt | Jan – Dec 2007 | DWTP | Membrane filtration | Culture | QIAamp DNA mini purification kit | TaqMan qPCR | ompW | - | - |
| Fang et al.^24^ | United States | 2012 – 2014 | Seawater | - | Culture | Boiling method | RT-qPCR | ompU, toxR, hlyA, rtxA | - | Amoxicillin-clavulanic acid (14%), Cephalothin (9%), Tetracycline (11%), Kanamycin (3%), Streptomycin (11%), Amikacin (11%) |
| Faouzi et al.^25^ | Morocco | Feb – August 2020 | Irrigation water | - | Culture | - | - | - | - | - |
| Ferdous et al.^26^ | Bangladesh | Sep 2014 – Oct 2015 | Drinking water | - | - | TBC | RT-qPCR | ctxA, rfbO1, rfbO139, cep, ace, msh1, stn/sto, rtxA, toxR, tcpI, hlyA, ompU, nag-st, rtxC, hap, chxA, vcsC2, vcsN2, vopF, vasK, vasA, vasH | O1, O139, non-O1/non-O139 | - |
| Ferguson et al.^27^ | Bangladesh | Jul – Aug 2009 | Tubewells | Membrane filtration | - | Fast DNA Spin Kit for Soil | RT-qPCR | ompW | - | - |
| Ferna ́ndez-Delgado et al.^28^ | Venezuela | Oct 2011 – Mar 2012 | Lagoon | Membrane filtration | Culture, serological test | QIAamp DNA Mini Kit | RT-qPCR | - | O1, O139 | - |
| Fraga et al.^29^ | Argentina | Jan 2003 – Jun 2005 | Rivers | Membrane filtration | Culture, serological test |  | RT-qPCR | hlyA, rtxA, toxR, stn-sto | Non-O1/non-O139 | Ampicillin (21.3% - 45.6%), Tetracycline (1.9%), Trimethoprim/Sulphamethoxazole (0.6%), Nitrofurantoin (3.9% - 7.3%) |
| George et al.^30^ | Bangladesh | Apr 2015 – June 2016 | Drinking water | Membrane filtration | Culture, serological test | - | - | ctxA | O1 | - |
| Gil et al.^31^ | Peru | Oct 1997 – Jun 2000 | Seawater | Membrane filtration | Culture, DFA | - | RT-qPCR | toxR, ctxA | O1, O22 | - |
| Goh et al.^32^ | Singapore | Apr 2013 – Jan 2014 | Coastal waters | Membrane filtration | Culture | Boiling method | RT-qPCR | - | - | - |
| Grothen et al.^33^ | Peru | - | River | Membrane filtration | - | - | RT-qPCR | ctxA | - | - |
| Halder et al.^34^ | India | Mar 2013 – Feb 2014 | River | - | Culture, serological test | - | RT-qPCR | hylA, rtxA, toxR, tcp, tlcR, toxT, RJ and LJ, and aldA | Non-O1/non-O139, O1 | - |
| Hosen et al.^35^ | Bangladesh |  | Pond, river | - | Culture | - | - | - | - | Cephalexin (83.33%), Vancomycin (100%), Penicillin (100%), Chloramphenicol (8.33%), Tetracycline (66.67%), Erythromycin (100%), Sulfamethoxazole (8.33%), Nalidixic acid (100%), Azithromycin (33.33%) |
| Hounmanou et al.^36^ | Tanzania | Jun – Dec 2007 | Lake | Membrane filtration | Culture | Maxwell DNA extraction system | RT-qPCR | ctxA | O1 | - |
| Islam et al.^37^ | Bangladesh | Mar – Aug 2009 | Ponds | - | Culture | - | - | - | Non-O1/non-O139 | - |
| Jesudason et al.^38^ | India | Apr 1997 – Mar 1998 | Surface water | Membrane filtration | Culture, biochemical tests, DFA test | - | - | - | O1 and O139 | - |
| Kaboré et al.^39^ | Burkina Faso | Apr – Jun 2014 | Rivers | - | Culture | Heating method | RT-qPCR | rfb, ctxA | Non-O1/non-O139 | - |
| Kachienga et al.^40^ | South Africa | Aug – Oct 2023 | Rivers | Membrane filtration | Culture, biochemical tests | ZymoBIOMICSTM DNA Miniprep Kit | RT-qPCR | ompW | - | - |
| Kaddumukasa et al.^41^ | Uganda | Sep 2009 – Aug 2010 | Lakes | Membrane filtration | Culture, biochemical tests | - | - | - | - | - |
| Kahler et al.^42^ | Haiti | Oct 2011 – Jan 2013 | Freshwater samples | Ultrafiltration | Culture | - | RT-qPCR | ompW, toxR, ctxA, | O1 and O139 | - |
| Keawvichit et al.^43^ | Thailand | Feb – Jul 2000 | Lagoon, irrigation water | Centrifugation | Culture, serological test | - | - | - | Non-O1/non-O139 | - |
| Kim et al.^44^ | Korea | Jan 2021 – Dec 2021 | Treated wastewater | Membrane filtration | FilmArray GI panel | - | - | - | - | - |
| Kirschner et al.^45^ | Austria | Oct 2016 – Jul 2017 | Bathing sites samples | Membrane filtration | Culture, MALDI-TOF | - | RT-qPCR | ompW | nonO1/nonO139 | - |
| Kokashvili et al.^46^ | Georgia | Jun 2006 – Oct 2008 | Surface water samples | Membrane filtration | Culture, biochemical tests, DFA test | AquaPure genomic DNA isolation kit | RT-qPCR, PCR/ESI-MS | - | O1 and O139 | - |
| Lee et al.^47^ | South Korea | Jan 2017 – Dec 2018 | Seawater | Membrane filtration | Culture | Heating method | RT-qPCR | hylA, ctxA | Non-O1, non-O139 | - |
| Lipp et al.^48^ | Peru | Nov 1998 – Dec 2000 | Seawater | Membrane filtration | - | CTAB and phenol-chloroformisopropyl alcohol. | RT-qPCR | ctxA | O1 | - |
| Luo et al.^49^ | China | May – Oct 2015 & 2016 | River | - | Culture, biochemical tests, serological test | DNeasy Blood & Tissue kit | RT-qPCR | ompW | non-O1/non-O139 | cefazolin (68.70%), ampicillin (47.83%), imipenem (27.83%), ciprofloxacin (9.57%), tetracycline (9.57%), cefoxitin (9.57%), chloramphenicol (7.83%), cefotaxime (1.74%), gentamicin (1.74%) |
| Malayil et al.^50^ | Georgia | Jun – Sep 2008 & Jan 2009 | Marine water | Membrane filtration | - | DNeasy Tissue Kit | RT-qPCR | ctxA | - | - |
| Matthews et al.^51^ | India | Apr 2014 – Mar 2015 | Stream | - | Culture, biochemical test, serological test | - | RT-qPCR | sodB, ctxAB | - | ampicillin (100%), ciprofloxacin (100%), tetracycline (100%), nalidixic acid (100%), gentamicin (100%), co-trimoxazole (100%) |
| Mishra et al.^52^ | India | Mar 2007 – Dec 2008 | Fresh water samples | Membrane filtration | Culture, biochemical test, serological test, DFA-DVC test | - | RT-qPCR | ompW | O1 | - |
| Mogessie et al.^53^ | Ethiopia | May – Jul 2023 | Drinking water, wastewater | Membrane filtration | Culture, serological test | - | - | - | O1 Hikojima, O1 Inaba | Amoxycillin (100%), Ampicillin (100%), Azithromycin (46.2%), Cefoxitin (53.8%), Ceftazidime (82.1%), Ceftriaxone (51.3%), Ciprofloxacin (23.1%), Meropenem (25.6%), Nalidixic acid (82.1%), Piperacillin/Tazobactam (5.1%), Tetracycline (64.1%), Trimethoprim/Sulphamethoxazole (69.2%) |
| Mok et al.^54^ | Korea | Jan – Dec 2017 | Seawater samples | - | Culture, biochemical tests, serological tests, | - | - | - | Non-O1/non-O139 | - |
| Momtaz et al.^55^ | Iran | Jul – Dec 2010 | Tap water, bottled mineral water | Membrane filtration | Culture | - | - | - | - | - |
| Moorkerjee et al.^56^ | India | Jan – Dec 2009 | River | Membrane filtration | Culture, biochemical test | - | RT-qPCR | tcpAEl Tor, ToxR, toxT, RJ & LJ, Zot | Non-O1/O139 | - |
| Moorkerjee et al. 2^57^ | India | Jan – Dec 2011 | River | - | Culture, serological test | Boiling method | RT-qPCR | ctx, tcp | O1 | - |
| Nayak et al.^58^ | India | Jul 2010 – Sep 2013 | Environmental water samples | - | Culture, biochemical and serological tests | - | MAMA and DMAMA PCR | ctxA, tcpA (El Tor), rfb (O1), toxR, El Tor, Classical and Haitian ctxB | O1, non-O1/ non-O139 | - |
| Ng et al.^59^ | Singapore | May – Jul 2016 | Ballast water and harbor water | - | Culture | Boiling method | RT-qPCR | ISR | - | - |
| Onyuka et al.^60^ | Kenya | - | Seawater | - | Culture, biochemical tests, serological test | - | - | - | O1 | Ampicillin (66.7%), Tetracycline (66.7%), Co-trimoxazole (66.7%) |
| Pal et al.^61^ | India | Jun 2017 – Mar 2020 | Freshwater samples | - | - | - | DMAMA-PCR | ctxB, tcpA, toxR, ompW, hly, ompU, rtx, rfbO1 | O1, O139, non-O1/non-O139 | - |
| Palit et al.^62^ | India | May 2007 – Nov 2008 | Potable water sources | Membrane filtration | Culture, serological test | - | RT-qPCR | ompW | O1, non-O1 | - |
| Potgieter et al.^63^ | South Africa | June – Nov 2016 | Rivers | Membrane filtration | Culture | - | RT-qPCR | - | - | - |
| Rafique et al.^64^ | Bangladesh | - | Stored drinking water, source water samples | Membrane filtration | Culture, serological test | - | DMAMA-PCR | rfbO1, ctxA, tcpA_ET_, ctxB | O1 | - |
| Rai et al.^65^ | Nepal | Jun 2014 | Sewage | - | Culture, serological test | - | - | - | O1 | - |
| Rasheed et al.^66^ | Pakistan | - | Drinking water samples | - | - | Ethanol precipitation method | RT-qPCR | - | - | - |
| Sacheli et al.^67^ | Belgium | May – Sep 2021 | Lakes | Membrane filtration | Culture, serological test, MALDI-TOF | - | - | - | Non-O1/non-O139 | - |
| Saima et al.^68^ | Bangladesh | Sep 2014 – Oct 2015 | Drinking water, groundwater | Membrane filtration | Culture | Boiling method | RT-qPCR | ompW | - | - |
| Saravanan et al.^69^ | India | - | River estuaries, river sediment | - | Culture, biochemical test | - | RT-qPCR | toxR | - | - |
| Schriewer et al. ^70^ | United States | Aug 2007 – Sep 2008 | Rivers and estuaries | Ultrafiltration | Culture, biochemical test | - | - | - | - | - |
| Shanan et al.^71^ | Sudan | - | Water reservoir, water tanks, lakes | - | - | Qiagen DNA mini kit | RT-qPCR | toxA | - | - |
| Shishir et al.^72^ | Bangladesh | - | Household water, pond sand filters, pond surface water | Membrane filtration | Culture, biochemical test, serological test | - | - | - | O1, non-O1/non-O139 | Co-trimoxazole (100%), erythromycin (100%), tetracycline (42%) |
| Singh and Lin^73^ | South Africa | Mar 2011 – Jan 2012 | River | Membrane filtration | - | - | - | - | - | - |
| Sirajul et al.^74^ | Bangladesh | Aug 2004 – Jan 2005 | Drinking water sources | Membrane filtration | Culture, biochemical test, serological test | - | - | - | O1, O139, non-O1/non-O139 | - |
| Sorensen et al.^75^ | Zambia | - | Groundwater | Membrane filtration | - | Power Water DNA isolation kit | RT-qPCR | - | - | - |
| Ssemanda et al.^76^ | Rwanda | Feb – Oct 2015 | Irrigation water | - | - | Power Water DNA isolation kit | RT-qPCR | - | - | - |
| Taviani et al.^77^ | Mozambique | Mar 2018 – Oct 2019 | DWTP | Membrane filtration | Culture, serological test | Bacterial cell lysate | RT-qPCR | ctx | Non-O1/non-O139 | Ampicillin (98.6%), Streptomycin (51%), Gentamicin (13%), Tetracycline (1.4%) |
| Teklehaimanot et al.^78^ | South Africa | Aug 2011 – May 2012 | Wastewater effluent and rivers | - | Culture, biochemical test | ZR Fungal/Bacterial DNA Kit | RT-qPCR | - | - | - |
| Thongchankaew et al.^79^ | Thailand | Jan – Dec 2008 | Lake | Membrane filtration | Culture | Qiagen DNA extraction kit | RT-qPCR | - | Non-O1/non-O139 | - |
| Torresi et al.^80^ | Italy | Aug 2010 – Aug 2011 | WWTP | Membrane filtration | Culture, serological test | - | RT-qPCR | toxR, hlyA | Non-O1/non-O139 | - |
| Wang et al.^81^ | China | 2018 – 2019 | River | Membrane filtration | Culture | - | RT-qPCR | - | non-O1/non-O139 | - |
| Waturangi et al.^82^ | Indonesia | Jun 2009 – May 2010 | Ice | - | Culture, biochemical test, serological test | Bacterial cell lysate | RT-qPCR | toxR, hlyA, ctxA, ompU | O1, non-O1/non-O139 | - |
| Whitehouse et al.^83^ | Georgia | Jul 2006 – Oct 2007 | Freshwater lakes and seawater | - | - | - | - | ctxA | - | - |
| Wongworapat et al.^84^ | Thailand | Feb – Jul 2000 | Lagoon, irrigation water, raw and treated wastewater | Centrifugation | Culture, biochemical tests, serological test | - | - | - | O139, Non-O1/non-O139, | - |
| Wu et al.^85^ | China | Jun – Aug 2016 | Groundwater samples | Membrane filtration | - | Fast DNA SPIN Kits for Soil | RT-qPCR | - | - | - |
| Yan et al.^86^ | China | Mar – Dec 2016 | Fish farm water, sediment | - | - | - | SN/T 101 1022-2010 method | - | - | - |
| Yue et al.^87^ | China | Jan 2008 – Dec 2009 | River estuary | - | Culture, biochemical test, serological test | genomic DNA purification kit | RT-qPCR | - | O1, O139 | - |

**Supplementary Table 2.** Meta-regression of factors affecting heterogeneity in the study

| **Covariates** | **Estimate** | **Standard Error** | **z-value** | **p-value** | **95% Confidence Interval** | |
| --- | --- | --- | --- | --- | --- | --- |
| **Water Environment** | | | | | | |
| Drinking Water (Intercept) | 0.1859 | 0.1234 | 1.5064 | 0.1320 | -0.0560 | 0.4277 |
| Untreated Sewage*** | 0.3818 | 0.1046 | 3.6518 | **0.0003** | 0.1769 | 0.5867 |
| Treated Sewage*** | 0.6590 | 0.1695 | 3.8888 | **0.0001** | 0.3269 | 0.9912 |
| Surface Water*** | 0.2763 | 0.0651 | 4.2441 | **<0.0001** | 0.1487 | 0.4039 |
| Groundwater | 0.1036 | 0.1106 | 0.9369 | 0.3488 | -0.1131 | 0.3203 |
| Others | 0.0846 | 0.0891 | 0.9497 | 0.3423 | -0.0900 | 0.2593 |
| **Continent** | | | | | | |
| Asia | -0.0890 | 0.0653 | -1.3630 | 0.1729 | -0.2170 | 0.0390 |
| Australia* | 0.6890 | 0.3032 | 2.2725 | **0.0231** | 0.0947 | 1.2832 |
| Europe | 0.0722 | 0.1483 | 0.4868 | 0.6264 | -0.2185 | 0.3629 |
| North America** | 0.3174 | 0.1179 | 2.6931 | **0.0071** | 0.0864 | 0.5484 |
| South America | -0.2216 | 0.1236 | -1.7935 | 0.0729 | -0.4638 | 0.0206 |
| **World Bank Country Classification** | | | | | | |
| Low-income economies | 0.1695 | 0.1260 | 1.3444 | 0.1788 | -0.0776 | 0.4165 |
| Lower-middle-income economies** | 0.2939 | 0.0962 | 3.0562 | **0.0022** | 0.1054 | 0.4824 |
| Upper-middle-income economies*** | 0.4566 | 0.1066 | 4.2846 | **<0.0001** | 0.2477 | 0.8740 |
| Unclassified | 0.2582 | 0.3142 | 0.8218 | 0.4112 | -0.3576 | 0.8740 |

**Supplementary Table 3**. Sensitivity Analysis of Pooled Prevalence

| **Excluded Study** | **Prevalence (%)** | **Lower CI** | **Upper CI** |
| --- | --- | --- | --- |
| Abana et al. | 36.69 | 31.85 | 41.66 |
| Abana et al. | 36.59 | 31.75 | 41.56 |
| Ahmad et al. | 36.57 | 31.74 | 41.53 |
| Ahmad et al. | 36.44 | 31.61 | 41.39 |
| Ahmad et al. | 36.38 | 31.56 | 41.33 |
| Ahmed et al. | 36.30 | 31.47 | 41.26 |
| Akoachere and Mbuntcha | 36.66 | 31.82 | 41.63 |
| Bauza et al. | 36.62 | 31.78 | 41.59 |
| Chaturongkasumrit et al. | 36.00 | 31.20 | 40.94 |
| Chomvarin et al. | 36.34 | 31.52 | 41.29 |
| El-Sayed et al. | 36.77 | 31.93 | 41.73 |
| El-Sayed et al. | 36.58 | 31.75 | 41.54 |
| Ferdous et al. | 36.68 | 31.77 | 41.72 |
| George et al. | 36.58 | 31.75 | 41.54 |
| George et al. | 36.47 | 31.64 | 41.43 |
| Mogessie et al. | 36.62 | 31.78 | 41.59 |
| Mogessie et al. | 36.46 | 31.63 | 41.42 |
| Momtaz et al. | 36.76 | 31.93 | 41.72 |
| Palit et al. | 36.69 | 31.84 | 41.67 |
| Rafique et al. | 36.54 | 31.70 | 41.50 |
| Rasheed et al. | 36.75 | 31.91 | 41.71 |
| Saima et al. | 36.68 | 31.74 | 41.56 |
| Shanan et al. | 36.77 | 31.95 | 41.72 |
| Shishir et al. | 36.57 | 31.74 | 41.54 |
| Sirajul et al. | 36.68 | 31.83 | 41.65 |
| Taviani et al. | 36.48 | 31.64 | 41.45 |
| Wongworapat et al. | 36.42 | 31.59 | 41.37 |
| Chomvarin et al. | 36.45 | 31.63 | 41.41 |
| Chomvarin et al. | 36.28 | 31.46 | 41.24 |
| Kim et al. | 36.53 | 31.70 | 37.82 |
| Mogessie et al. | 36.52 | 31.69 | 41.49 |
| Mogessie et al. | 36.51 | 31.68 | 41.48 |
| Rai et al. | 36.50 | 31.67 | 41.45 |
| Teklehaimanot et al. | 35.88 | 31.09 | 40.81 |
| Teklehaimanot et al. | 35.88 | 31.09 | 40.81 |
| Teklehaimanot et al. | 35.99 | 31.19 | 40.93 |
| Teklehaimanot et al. | 35.88 | 31.09 | 40.81 |
| Torresi et al. | 36.38 | 31.55 | 41.34 |
| Wongworapat et al. | 36.42 | 31.59 | 41.37 |
| Teklehaimanot et al. | 35.99 | 31.19 | 40.93 |
| Teklehaimanot et al. | 36.05 | 31.25 | 40.99 |
| Teklehaimanot et al. | 35.99 | 31.19 | 40.93 |
| Teklehaimanot et al. | 35.88 | 31.09 | 40.81 |
| Abana et al. | 36.19 | 31.38 | 41.14 |
| Akoachere and Mbuntcha | 36.30 | 31.48 | 41.25 |
| Alam et al. | 36.66 | 31.82 | 41.64 |
| Aulet et al. | 36.15 | 31.34 | 41.10 |
| Bahk et al. | 36.57 | 31.73 | 41.55 |
| Bhandari et al. | 36.07 | 31.26 | 41.01 |
| Bisimwa et al. | 36.16 | 31.49 | 40.95 |
| Bliem et al. | 36.28 | 31.46 | 41.24 |
| Böer et al. | 36.76 | 31.93 | 41.72 |
| Bwire et al. | 36.59 | 31.75 | 41.56 |
| Bwire et al. | 36.65 | 31.82 | 41.62 |
| Bwire et al. | 36.70 | 31.87 | 41.67 |
| Chandran et al. | 36.37 | 31.55 | 41.34 |
| Chandran et al. | 36.33 | 31.51 | 41.29 |
| Chigbu and Iroegbu | 36.35 | 31.53 | 41.31 |
| Chigbu and Iroegbu | 36.33 | 31.50 | 41.29 |
| Chomvarin et al. | 36.28 | 31.46 | 41.24 |
| Coly et al. | 36.68 | 31.84 | 41.64 |
| Dickinson et al. | 36.37 | 31.54 | 41.33 |
| Dickinson et al. | 36.57 | 31.73 | 41.54 |
| du Preez et al. | 36.39 | 31.55 | 41.35 |
| Dumontet et al. | 36.57 | 31.72 | 41.54 |
| El-Sayed et al. | 36.58 | 31.75 | 41.54 |
| Fang et al. | 36.30 | 31.47 | 41.26 |
| Ferna ́ndez-Delgado et al. | 36.50 | 31.67 | 41.46 |
| Fraga et al. | 36.41 | 31.56 | 41.40 |
| Gil et al. | 36.57 | 31.73 | 41.55 |
| Goh et al. | 36.60 | 31.75 | 41.57 |
| Grothen et al. | 36.58 | 31.75 | 41.54 |
| Halder et al. | 36.21 | 31.40 | 41.16 |
| Hosen et al. | 36.37 | 31.55 | 41.32 |
| Hosen et al. | 36.68 | 31.56 | 41.33 |
| Hounmanou et al. | 36.69 | 31.85 | 41.66 |
| Islam et al. | 35.92 | 31.13 | 40.84 |
| Islam et al. | 35.92 | 31.13 | 40.84 |
| Islam et al. | 36.33 | 31.51 | 41.29 |
| Islam et al. | 35.91 | 31.11 | 40.84 |
| Islam et al. | 36.45 | 31.62 | 41.41 |
| Islam et al. | 36.28 | 31.46 | 41.23 |
| Islam et al. | 36.51 | 31.68 | 41.47 |
| Islam et al. | 36.41 | 31.58 | 41.37 |
| Jesudason et al. | 36.13 | 31.31 | 41.07 |
| Kaboré et al. | 36.40 | 31.57 | 41.36 |
| Kachienga et al. | 36.34 | 31.51 | 41.29 |
| Kaddumukasa et al. | 36.10 | 31.30 | 41.03 |
| Kahler et al. | 35.89 | 31.09 | 40.82 |
| Kahler et al. | 35.89 | 31.09 | 40.82 |
| Kahler et al. | 35.89 | 31.09 | 40.82 |
| Kahler et al. | 35.89 | 31.09 | 40.82 |
| Keawvichit et al. | 36.32 | 31.50 | 41.27 |
| Kirschner et al. | 36.23 | 31.41 | 41.18 |
| Kokashvili et al. | 36.10 | 31.45 | 40.88 |
| Lee et al. | 36.55 | 31.52 | 41.71 |
| Lee et al. | 36.49 | 31.47 | 41.65 |
| Lipp et al. | 36.30 | 31.48 | 41.26 |
| Luo et al. | 36.32 | 31.49 | 41.28 |
| Malayil et al. | 36.37 | 31.55 | 41.34 |
| Matthews et al. | 36.20 | 31.39 | 41.15 |
| Mishra et al. | 36.37 | 31.53 | 41.35 |
| Mok et al. | 36.79 | 31.96 | 41.74 |
| Moorkerjee et al. | 36.41 | 31.58 | 41.38 |
| Moorkerjee et al. 2 | 36.39 | 31.55 | 41.36 |
| Onyuka et al. | 36.68 | 31.85 | 41.65 |
| Pal et al. 2 | 36.72 | 32.05 | 41.51 |
| Potgieter et al. | 36.18 | 31.37 | 41.13 |
| Rafique et al. | 36.47 | 31.63 | 41.43 |
| Sacheli et al. | 36.50 | 31.67 | 41.46 |
| Saravanan et al. | 36.48 | 31.65 | 41.44 |
| Schriewer et al. | 36.34 | 31.51 | 41.31 |
| Shanan et al. | 36.62 | 31.78 | 41.58 |
| Shishir et al. | 36.42 | 31.59 | 41.39 |
| Singh and Lin | 35.86 | 31.07 | 40.79 |
| Taviani et al. | 35.87 | 31.08 | 40.80 |
| Teklehaimanot et al. | 35.93 | 31.18 | 40.82 |
| Thongchankaew et al. | 36.59 | 31.76 | 41.55 |
| Wang et al. | 36.63 | 31.79 | 41.60 |
| Whitehouse et al. | 36.37 | 31.53 | 41.35 |
| Yue et al. | 36.72 | 31.89 | 41.68 |
| Abana et al. | 36.59 | 31.75 | 41.55 |
| Akoachere and Mbuntcha | 36.46 | 31.61 | 41.44 |
| Alaoui et al. | 36.11 | 31.30 | 41.06 |
| Bwire et al. | 36.52 | 31.68 | 41.48 |
| Ferguson et al. | 36.63 | 31.79 | 41.59 |
| Saima et al. | 36.64 | 31.79 | 41.62 |
| Sorensen et al. | 36.37 | 31.54 | 41.33 |
| Wu et al. | 36.44 | 31.60 | 41.41 |
| Böer et al. | 36.72 | 31.89 | 41.69 |
| Bwire et al. | 36.40 | 31.58 | 41.36 |
| Chaturongkasumrit et al. | 36.18 | 31.36 | 41.12 |
| Chomvarin et al. | 36.28 | 31.46 | 41.23 |
| Faouzi et al. | 36.22 | 31.40 | 41.16 |
| Keawvichit et al. | 36.37 | 31.55 | 41.32 |
| Matthews et al. | 36.38 | 31.55 | 41.34 |
| Nayak et al. | 36.76 | 31.96 | 41.69 |
| Nayak et al. | 36.76 | 31.97 | 41.69 |
| Ng et al. | 36.50 | 31.67 | 41.45 |
| Saravanan et al. | 36.50 | 31.67 | 41.45 |
| Shishir et al. | 36.47 | 31.64 | 41.43 |
| Ssemanda et al. | 36.58 | 31.75 | 41.54 |
| Waturangi et al. | 36.41 | 31.58 | 41.38 |
| Wongworapat et al. | 36.47 | 31.64 | 41.43 |
| Yan et al. | 36.64 | 31.72 | 41.70 |
| Yan et al. | 36.67 | 31.77 | 41.70 |

**Supplementary Table 4**. Main reasons of exclusion of eligible studies

| **No** | **Author, Year** | **Title** | **Reason for exclusion** |
| --- | --- | --- | --- |
| 1 | Abdulaziz, 2023 | Molecular Detection and Distribution of Six Medically Important Vibrio spp. in Selected Freshwater and Brackish Water Resources in Eastern Cape Province, South Africa | No prevalence data |
| 2 | Abia, 2016 | Competitive Survival of Escherichia coli, Vibrio cholerae, Salmonella typhimurium and Shigella dysenteriae in Riverbed Sediments | No prevalence data |
| 3 | Abia, 2017 | Riverbed Sediments as Reservoirs of Multiple Vibrio cholerae Virulence-Associated Genes: A Potential Trigger for Cholera Outbreaks in Developing Countries | No prevalence data |
| 4 | Abioye, 2021 | Molecular Detection and Distribution of Six Medically Important Vibrio spp. in Selected Freshwater and Brackish Water Resources in Eastern Cape Province, South Africa | No prevalence data |
| 5 | Abioye, 2023 | Occurrence of virulence determinants in vibrio cholerae, vibrio mimicus, vibrio alginolyticus, and vibrio parahaemolyticus isolates from important water resources of Eastern Cape, South Africa | No prevalence data |
| 6 | Aboh, 2015 | Microbiological assessment of well waters in Samaru, Zaria, Kaduna, State, Nigeria | VC not detected |
| 7 | Adesiyan, 2022 | Incidence of antibiotic resistance genotypes of Vibrio species recovered from selected freshwaters in Southwest Nigeria. | No prevalence data |
| 8 | Adesiyan, 2021 | Occurrence and antibiogram signatures of some Vibrio species recovered from selected rivers in South West Nigeria | No prevalence data |
| 9 | Agboola, 2023 | Distribution and antibiogram of Vibrio species from hospital wastewater in Southwest, Nigeria | No prevalence data |
| 10 | Ahmad, 2012 | Role of hospital effluents in the contribution of antibiotics and antibiotic resistant bacteria to the aquatic environment | No prevalence data |
| 11 | Ahogle, 2024 | Bacterial hazards in urban stream irrigation in peri-urban interface of Nairobi-Machakos counties, Kenya | No prevalence data |
| 12 | Al-Bayatti, 2012 | Bacteriological and physicochemical studies on tigris river near the water purification stations within baghdad province | No prevalence data |
| 13 | Ali, 2023 | Evidential role of municipal solid waste and liquid effluent on environment and public health | <10 samples |
| 14 | Behera, 2023 | Anthropogenic impact and antibiotic resistance among the indicator and pathogenic bacteria from several industrial and sewage discharge points along the coast from Pydibhimavaram to Tuni, East Coast of India | No prevalence data |
| 15 | Biswas, 2014 | Contaminated Pond Water Favors Cholera Outbreak at Haibatpur Village, Purba Medinipur District, West Bengal, India | <10 samples |
| 16 | Bonadonna, 2019 | Enteric viruses, somatic coliphages and Vibrio species in marine bathing and non-bathing waters in Italy | Unspecified prevalence data |
| 17 | Bonyadian, 2018 | PCR detection of Vibrio cholerae, Escherichia coli, and Salmonella sp. from bottled drinking water in Iran | VC not detected |
| 18 | Daboul, 2020 | Characterization of Vibrio cholerae isolates from freshwater sources in northwest Ohio | No prevalence data |
| 19 | De Guzman, 2015 | Availability of safe drinking-water: the answer to cholera outbreak? Nabua, Camarines Sur, Philippines, 2012 | <10 samples |
| 20 | Dheenan, 2014 | Spatial variation of physicochemical and bacteriological parameters elucidation with GIS in Rangat Bay, Middle Andaman, India | No prevalence data |
| 21 | Di, 2017 | Season-specific occurrence of potentially pathogenic Vibrio spp. on the southern coast of South Korea | No prevalence data |
| 22 | Djaouda, 2013 | Survival and Growth of Vibrio cholerae, Escherichia coli, and Salmonella Spp. in Well Water Used for Drinking Purposes in Garoua (North Cameroon). | No prevalence data |
| 23 | Dobbs, 2013 | Pandemic Serotypes of Vibrio cholerae Isolated from Ships' Ballast Tanks and Coastal Waters: Assessment of Antibiotic Resistance and Virulence Genes (tcpA and ctxA) | No prevalence data |
| 24 | Eja, 2008 | Seasonal occurrence of vibrios in water and shellfish obtained from the Great Kwa River estuary, Calabar, Nigeria | No prevalence data |
| 25 | Elhadi, 2013 | Occurrence of potentially human pathogenic Vibrio species in the coastal water of the Eastern Province of Saudi Arabia | Not accessible |
| 26 | Esteves, 2015 | Rapid Proliferation of Vibrio parahaemolyticus, Vibrio vulnificus, and Vibrio cholerae during Freshwater Flash Floods in French Mediterranean Coastal Lagoons | No prevalence data |
| 27 | Eurien, 2021 | Cholera outbreak caused by drinking unprotected well water contaminated with faeces from an open storm water drainage: Kampala City, Uganda, January 2019 | <10 samples |
| 28 | Eyisi, 2013 | Distribution of Vibrio species in shellfish and water samples collected from the Atlantic coastline of South-East Nigeria | No prevalence data |
| 29 | Farouk, 2020 | Environmental studies on water quality, plankton and bacterial community in Mariout Lake, Egypt | No prevalence data |
| 30 | Fernández-Delgado, 2009 | Vibrio cholerae non-O1, non-O139 associated with seawater and plankton from coastal marine areas of the Caribbean Sea | No prevalence data |
| 31 | Ford, 2020 | Isolation and characterization of potentially pathogenic Vibrio species in a temperate, higher latitude hotspot | No prevalence data |
| 32 | Fri, 2017 | Occurrence of virulence genes associated with human pathogenic vibrios isolated from two commercial Dusky Kob (Argyrosmus japonicus) farms and kareiga estuary in the Eastern Cape Province, South Africa | VC not detected |
| 33 | Fu, 2019 | A human intestinal infection caused by a novel non-O1/O139 Vibrio cholerae genotype and its dissemination along the river | <10 samples |
| 34 | Gdoura, 2016 | Molecular Detection of the Three Major Pathogenic Vibrio Species from Seafood Products and Sediments in Tunisia Using Real-Time PCR | VC not detected |
| 35 | Gerokomou, 2011 | Physical, chemical and microbiological quality of ice used to cool drinks and foods in Greece and its public health implications | VC not detected |
| 36 | Ginn, 2021 | Detection and Quantification of Enteric Pathogens in Aerosols near Open Wastewater Canals in Cities with Poor Sanitation | VC not detected |
| 37 | Griffitt, 2013 | Abundance and Distribution of Vibrio cholerae, V. parahaemolyticus, and V. vulnificus Following a Major Freshwater Intrusion into the Mississippi Sound | No prevalence data |
| 38 | Guardiola-Avila, 2018 | Isolation and identification of Vibrio species in the Rio Bravo/Grande and water bodies from Reynosa, Tamaulipas | No prevalence data |
| 39 | Hammer, 2019 | Metagenomic profiling of microbial pathogens in the little bighorn river, Montana | No prevalence data |
| 40 | Haque, 2019 | Assessment of physicochemical and bacteriological parameters in surface water of Padma River, Bangladesh | No prevalence data |
| 41 | Hill, 2011 | Toxigenic Vibrio Cholerae O1 in water and seafood, Haiti | <10 samples |
| 42 | Hiruy, 2022 | Spatiotemporal variation in urban wastewater pollution impacts on river microbiomes and associated hazards in the Akaki catchment, Addis Ababa, Ethiopia | No prevalence data |
| 43 | Håkonsholm, 2020 | Vibrios from the Norwegian marine environment: Characterization of associated antibiotic resistance and virulence genes | No prevalence data |
| 44 | Hoorzook, 2021 | Soul of the Jukskei River: The Extent of Bacterial Contamination in the Jukskei River in Gauteng Province, South Africa | <10 samples |
| 45 | Izumiya, 2017 | A double-quadratic model for predicting Vibrio species in water environments of Japan | No prevalence data |
| 46 | Jahid, 2013 | Role of polyphosphate kinase gene (ppk) for survival of Vibrio cholerae ol in surface water of Bangladesh | Not accessible |
| 47 | Jasmine, 2020 | Bacteriological quality assessment of groundwater and surface water in Chennai | No prevalence data |
| 48 | Jeamsripong, 2022 | Molecular Epidemiology of Antimicrobial Resistance and Virulence Profiles of Escherichia coli, Salmonella spp., and Vibrio spp. Isolated from Coastal Seawater for Aquaculture | No prevalence data |
| 49 | Kaponda, 2019 | Drinking water quality and human dimensions of cholera patients to inform evidence-based prevention investment in Karonga District, Malawi | No prevalence data |
| 50 | Khalil, 2014 | Seasonal bacteriological and physico-chemical analysis of Lake Timsah, Ismailia, Egypt | No prevalence data |
| 51 | Khouadja, 2014 | Occurrence of virulence genes among Vibrio cholerae and Vibrio parahaemolyticus strains from treated wastewaters | No prevalence data |
| 52 | Kirchberger, 2016 | A small number of phylogenetically distinct clonal complexes dominate a coastal Vibrio cholerae population | No prevalence data |
| 53 | Kokashvili, 2013 | Comparative phenotypic characterization of Vibrio cholerae isolates collected from aquatic environments of Georgia. | Not accessible |
| 54 | Kurpas, 2021 | First report of the presence of Vibrio vulnificus in the Gulf of Gdansk | Incomplete identification |
| 55 | Kwesiga, 2017 | A prolonged, community-wide cholera outbreak associated with drinking water contaminated by sewage in Kasese District, western Uganda | No prevalence data |
| 56 | Leard, 2023 | Environmental Drivers of Vibrio cholerae Abundances in Mobile Bay, Alabama. | No prevalence data |
| 57 | Lenglet, 2010 | A cholera epidemic in Sekong province, Lao people's Democratic republic, December 2007-January 2008 | No prevalence data |
| 58 | Li, 2015 | Characterization of environmental Vibrio cholerae serogroups O1 and O139 in the Pearl River Estuary, China | Incomplete prevalence data |
| 59 | Lofti, 2020 | Assessment of microbiological quality of groundwater in the saïs plain (Morocco) | No prevalence data |
| 60 | Madhusudana, 2013 | Detection of ctx gene positive non-O1/non-O139 V. cholerae in shrimp aquaculture environments | <10 samples |
| 61 | Madoroba, 2010 | Prevalence of vibrio cholerae in rivers of mpumalanga province, south africa as revealed by polyphasic characterization | No prevalence data |
| 62 | Maje, 2020 | Characterisation of Vibrio Species from Surface and Drinking Water Sources and Assessment of Biocontrol Potentials of Their Bacteriophages | No prevalence data |
| 63 | Manegabe, 2017 | Antibiotic resistance and tolerance to heavy metals demonstrated by environmental pathogenic bacteria isolated from the Kahwa River, Bukavu Town, Democratic Republic of the Congo | No prevalence data |
| 64 | Mariita, 2009 | Usefulness of faecal Streps as indicator of presence of Salmonella sp. and Vibrio cholerae in sewage effluents | <10 samples |
| 65 | Martinelli, 2016 | Are natural reservoirs important for cholera surveillance? The case of an outbreak in a Brazilian estuary | <10 samples |
| 66 | Martin, 2013 | Microbial analysis in Tamiraparani and Palayaru river in Kanyakumari District | Not accessible |
| 67 | Miyagi, 2003 | Survey of Vibrio cholerae O1 and its survival over the winter in marine water of Port of Osaka. | Incomplete prevalence data |
| 68 | Mousa, 2024 | Bacterial Diversity in Al-Asfar Lake, Al Ahsa Oasis, Saudi Arabia | No prevalence data |
| 69 | Nagvenkar, 2009 | Abundance of sewage-pollution indicator and human pathogenic bacteria in a tropical estuarine complex | No prevalence data |
| 70 | Nasreen, 2022 | Dynamic Subspecies Population Structure of Vibrio cholerae in Dhaka, Bangladesh | No prevalence data |
| 71 | Nigro, 2011 | Temporal and spatial variability in culturable pathogenic vibrio spp. in Lake Pontchartrain, Louisiana, following hurricanes katrina and rita | No prevalence data |
| 72 | Nyati, 2004 | Evaluation of the microbial quality of water supplies to municipal, mining and squatter communities in the Bindura urban area of Zimbabwe | VC not detected |
| 73 | Ocholi, 2024 | Bacteriological quality of drinking water from wells located near municipal solid waste dumps and liquid waste sites in Zaria, Kaduna State, Nigeria | Not accessible |
| 74 | Okello, 2019 | A cholera outbreak caused by drinking contaminated river water, Bulambuli District, Eastern Uganda, March 2016 | <10 samples |
| 75 | O.m.al-Dahmoshi, 2020 | Bacteriological study of municipal water discharged in al-kufa river, Najaf, Iraq | No prevalence data |
| 76 | Onohuean, 2021 | Epidemiologic potentials and correlational analysis of Vibrio species and virulence toxins from water sources in greater Bushenyi districts, Uganda | No prevalence data |
| 77 | Ortiz-Carrillo, 2015 | Diversity of Vibrio spp in karstic coastal marshes in the Yucatan Peninsula | No prevalence data |
| 78 | Osunlo, 2015 | Distribution and public health significance of vibrio pathogens recovered from selected treated effluents in the eastern cape province, south africa | No prevalence data |
| 79 | Ouansafi, 2022 | Treated wastewater irrigation of tomato: Effects on crop production, and on physico-chemical properties, SDH activity and microbiological characteristics of fruits | No prevalence data |
| 80 | Oyedeji, 2013 | Molecular characterization of the circulating strains of Vibrio cholerae during 2010 cholera outbreak in Nigeria. | <10 samples |
| 81 | Pal, 2015 | Altered El Tor Vibrio cholerae O1 caused outbreak of cholera in the southern part of Odisha, India during 2011 | Not accessible |
| 82 | Prasanthan, 2011 | Influence of abiotic environmental factors on the abundance and distribution of Vibrio species in coastal waters of Kerala, India | Not accessible |
| 83 | Puto, 2019 | Impact of pathogenic microbes of water and mollusca Mytilus galloprovincialis in the butrinti lake | Not accessible |
| 84 | Rai, 2012 | Study of medically important Vibrios in the sewage of Katmandu Valley, Nepal. | Not accessible |
| 85 | Rajendran, 2016 | Bacteriological analysis of water samples from Tsunami hit coastal areas of Kanyakumari District, Tamil Nadu | VC not detected |
| 86 | Rashid, 2013 | Detection of Vibrio cholerae in environmental waters including drinking water reservoirs of Azerbaijan | No prevalence data |
| 87 | Reddy, 2022 | A study on drinking water quality in different income groups of Vizianagaram region of India | No prevalence data |
| 88 | Reethy, 2021 | Characterization of V. cholerae O1 biotype El Tor serotype Ogawa possessing the ctxB gene of the classical biotype isolated from well water associated with the cholera outbreak in Kerala, South India | No prevalence data |
| 89 | Rehm, 2023 | First report on the occurrence of Vibrio cholerae nonO1/nonO139 in natural and artificial lakes and ponds in Serbia: Evidence for a long-distance transfer of strains and the presence of Vibrio paracholerae | No prevalence data |
| 90 | Righetto, 2015 | Detection of Vibrio cholerae O1 and O139 in environmental waters of rural Bangladesh: A flow-cytometry-based field trial | No prevalence data |
| 91 | Ristori, 2007 | Pathogenic bacteria associated with oysters (Crassostrea brasiliana) and estuarine water along the south coast of Brazil | VC not detected |
| 92 | Samie, 2012 | Diversity and antibiograms of bacterial organisms isolated from samples of household drinking-water consumed by HIV-positive individuals in rural ettings,South Africa | VC not detected |
| 93 | Schauer, 2015 | Dynamics of Vibrio cholerae abundance in Austrian saline lakes, assessed with quantitative solid-phase cytometry | No prevalence data |
| 94 | Schets, 2011 | Potentially human pathogenic vibrios in marine and fresh bathing waters related to environmental conditions and disease outcome | No prevalence data |
| 95 | Shahcheraghi, 2009 | Transmission of Vibrio cholera O1 serotype Inaba in a rural area of Qazvin, Iran associated with drinking water | Not accessible |
| 96 | Shar, 2010 | Seasonal variation of vibrio cholerae and vibrio mimicus in freshwater environment | No prevalence data |
| 97 | Shartooh, 2017 | Environmental and microbial investigation on the dredged sediment soil of diyala river in iraq | Not accessible |
| 98 | Sheikh, 2012 | Identification of Vibrio cholerae pathogenicity island (ctxA, OmpW and tcpA) in non-O139 and non-O1 V. cholerae strains isolated from Karun River in Ahvaz, Iran | No prevalence data |
| 99 | Siboni, 2016 | Spatiotemporal dynamics of Vibrio spp. within the Sydney Harbour estuary | No prevalence data |
| 100 | Sá, 2012 | Vibrio cholerae O1 from superficial water of the Tucunduba Stream, Brazilian Amazon | <10 samples |
| 101 | Sojka, 2022 | Recreational pools filled with mineralized thermal water are potential reservoirs of pathogenic Vibrio spp. | Not accessible |
| 102 | Sulca, 2018 | Antimicrobial resistance not related to 1,2,3 integrons and Superintegron in Vibrio spp. isolated from seawater sample of Lima (Peru) | No prevalence data |
| 103 | Sultana, 2024 | Isolation and Identification of Vibrio Species from Different Types of Water Sources Along with Their Drug Susceptible Pattern | <10 samples |
| 104 | Tall, 2012 | Real-time PCR optimization to identify environmental Vibrio spp. strains. | No prevalence data |
| 105 | Tambekar, 2014 | Physico-chemical and microbial analysis of certain water sources and industrial waste water samples in chandrapur district, Maharastra, India | Not accessible |
| 106 | Taneja, 2011 | Fecal contamination of drinking water supplies in and around chandigarh and correlation with acute gastroenteritis | Not accessible |
| 107 | Tey, 2015 | Occurrence of Vibrio parahaemolyticus, Vibrio cholerae, and Vibrio vulnificus in the Aquacultural Environments of Taiwan | No prevalence data |
| 108 | Uchiyama, 2015 | A study on the existence of Vibrio cholerae non-O1 in the river | No prevalence data |
| 109 | Vezzulli, 2021 | Aquatic reservoir of Vibrio cholerae in an African Great Lake assessed by large scale plankton sampling and ultrasensitive molecular methods | <10 samples |
| 110 | Vezzulli, 2009 | Benthic ecology of Vibrio spp. and pathogenic Vibrio species in a coastal Mediterranean environment (La Spezia Gulf, Italy) | No prevalence data |
| 111 | Vincy, 2017 | Prevalence of indicator and pathogenic bacteria in a tropical river of Western Ghats, India | VC not detected |
| 112 | Vozárová, 2024 | Occurrence, isolation, and identification of emergent pathogens of taxon Vibrio from the Danube river in Bratislava city, Slovakia | No prevalence data |
| 113 | Xaba, 2023 | Impact of Poor Stream Conditions on the Health of Stream Workers in Umlazi, KwaZulu-Natal, South Africa | Not accessible |
| 114 | Yam, 2000 | Abundance of clinical enteric bacterial pathogens in coastal waters and shellfish | VC not detected |
| 115 | Yongyod, 2023 | Microbiological Quality and Sanitation of Food Stalls and Drinking Water Vending Machines | VC not detected |
| 116 | Zahid, 2009 | Prevalence of multiple antibiotic resistant bacteria and chromosomal determinants in surface water of Bangladesh | No prevalence data |

Supplementary Table 5. Risk of Bias Assessment

| **Publication** | **Was the study's target population a close representation of the national population in relation to vibro cholera prevalence** | **Was the sampling frame a true or close representation of the samples** | **Was some form of random selection used to select the samples** | **Did the authors calculate and respect the expected sample size** | **Was a defined water environment used in the study** | **Was the vibro cholera detection assay shown to have validity and relaibility** | **Was one mode of data collection used for all samples** | **Was the length of the study period ≥ 1 year** | **Were the numerator(s) and denominator(s) for the prevalence of Vibro cholera reported** | **Risk of bias** |
| --- | --- | --- | --- | --- | --- | --- | --- | --- | --- | --- |
| Abana et al. | 1 | 1 | 1 | 0 | 1 | 1 | 1 | 0 | 1 | Low |
| Ahmad et al. | 0 | 1 | 1 | 0 | 1 | 1 | 1 | 0 | 1 | Moderate |
| Ahmed et al. | 0 | 0 | 1 | 0 | 1 | 1 | 1 | 0 | 0 | Moderate |
| Akoachere and Mbuntcha | 1 | 1 | 1 | 0 | 1 | 1 | 1 | 0 | 1 | Low |
| Alam et al. | 0 | 0 | 1 | 0 | 1 | 1 | 1 | 1 | 1 | Moderate |
| Alaoui et al. | 0 | 1 | 1 | 0 | 1 | 1 | 1 | 1 | 0 | Moderate |
| Aulet et al. | 0 | 1 | 0 | 0 | 1 | 1 | 1 | 1 | 1 | Moderate |
| Bahk et al. | 0 | 1 | 0 | 0 | 1 | 1 | 1 | 1 | 1 | Moderate |
| Bauza et al. | 1 | 1 | 0 | 0 | 1 | 1 | 1 | 1 | 0 | Moderate |
| Bhandari et al. | 1 | 1 | 1 | 1 | 0 | 1 | 1 | 0 | 1 | Low |
| Bisimwa et al. | 0 | 1 | 0 | 0 | 1 | 1 | 1 | 1 | 0 | Moderate |
| Bliem et al. | 0 | 1 | 0 | 0 | 1 | 1 | 1 | 0 | 1 | Moderate |
| Böer et al. | 0 | 1 | 0 | 0 | 1 | 1 | 1 | 1 | 1 | Moderate |
| Bwire et al. | 1 | 1 | 0 | 0 | 1 | 1 | 1 | 1 | 1 | Low |
| Chandran et al. | 0 | 1 | 0 | 0 | 1 | 1 | 1 | 1 | 1 | Moderate |
| Chaturongkasumrit et al. | 0 | 1 | 0 | 0 | 1 | 1 | 1 | 0 | 1 | Moderate |
| Chigbu and Iroegbu | 1 | 1 | 0 | 0 | 1 | 1 | 1 | 0 | 1 | Low |
| Chomvarin et al. | 1 | 1 | 1 | 0 | 1 | 1 | 1 | 1 | 1 | Low |
| Coly et al. | 0 | 1 | 0 | 0 | 1 | 1 | 1 | 1 | 0 | Moderate |
| Dickinson et al. | 0 | 1 | 1 | 0 | 1 | 1 | 1 | 0 | 1 | Moderate |
| du Preez et al. | 1 | 1 | 0 | 0 | 1 | 1 | 1 | 1 | 1 | Low |
| Dumontet et al. | 0 | 1 | 0 | 0 | 1 | 1 | 1 | 1 | 1 | Moderate |
| El-Sayed et al. | 0 | 1 | 1 | 0 | 1 | 1 | 1 | 0 | 1 | Moderate |
| Fang et al. | 0 | 1 | 0 | 0 | 0 | 1 | 1 | 1 | 0 | Moderate |
| Faouzi et al. | 0 | 1 | 0 | 0 | 0 | 1 | 1 | 0 | 1 | Moderate |
| Ferdous et al. | 0 | 1 | 0 | 0 | 1 | 1 | 1 | 1 | 1 | Moderate |
| Ferguson et al. | 0 | 1 | 0 | 0 | 1 | 1 | 1 | 0 | 0 | Moderate |
| Ferna ́ndez-Delgado et al. | 0 | 1 | 0 | 0 | 0 | 1 | 1 | 0 | 1 | Moderate |
| Fraga et al. | 0 | 1 | 0 | 0 | 1 | 1 | 1 | 1 | 1 | Moderate |
| George et al. | 1 | 1 | 0 | 0 | 0 | 1 | 1 | 1 | 1 | Moderate |
| Gil et al. | 0 | 1 | 0 | 0 | 0 | 0 | 1 | 1 | 1 | Moderate |
| Goh et al. | 0 | 1 | 0 | 0 | 1 | 1 | 1 | 0 | 1 | Moderate |
| Grothen et al. | 0 | 1 | 0 | 0 | 0 | 1 | 1 | 0 | 1 | Moderate |
| Halder et al. | 0 | 1 | 0 | 0 | 0 | 1 | 1 | 1 | 1 | Moderate |
| Hosen et al. | 1 | 1 | 0 | 0 | 1 | 1 | 1 | 0 | 1 | Moderate |
| Hounmanou et al. | 0 | 1 | 0 | 0 | 1 | 1 | 1 | 0 | 1 | Moderate |
| Islam et al. | 0 | 1 | 0 | 0 | 1 | 1 | 1 | 0 | 1 | Moderate |
| Jesudason et al. | 0 | 1 | 0 | 0 | 1 | 1 | 1 | 1 | 0 | Moderate |
| Kaboré et al. | 0 | 1 | 0 | 0 | 1 | 1 | 1 | 0 | 1 | Moderate |
| Kachienga et al. | 0 | 1 | 0 | 0 | 1 | 1 | 1 | 0 | 1 | Moderate |
| Kaddumukasa et al. | 0 | 1 | 0 | 0 | 1 | 1 | 1 | 1 | 1 | Moderate |
| Kahler et al. | 1 | 1 | 1 | 0 | 1 | 1 | 1 | 1 | 1 | Low |
| Keawvichit et al. | 0 | 1 | 0 | 0 | 1 | 1 | 1 | 0 | 0 | Moderate |
| Kim et al. | 0 | 1 | 0 | 0 | 1 | 1 | 1 | 0 | 1 | Moderate |
| Kirschner et al. | 0 | 1 | 0 | 0 | 1 | 1 | 1 | 1 | 1 | Moderate |
| Kokashvili et al. | 0 | 1 | 0 | 0 | 1 | 1 | 1 | 1 | 1 | Moderate |
| Lee et al. | 0 | 1 | 0 | 0 | 1 | 1 | 1 | 1 | 1 | Moderate |
| Lipp et al. | 1 | 1 | 0 | 0 | 1 | 1 | 1 | 1 | 1 | Low |
| Luo et al. | 0 | 1 | 0 | 0 | 1 | 1 | 1 | 1 | 1 | Moderate |
| Malayil et al. | 0 | 1 | 0 | 0 | 0 | 1 | 1 | 0 | 1 | Moderate |
| Matthews et al. | 0 | 1 | 0 | 0 | 1 | 1 | 1 | 1 | 1 | Moderate |
| Mishra et al. | 1 | 1 | 0 | 0 | 1 | 1 | 1 | 1 | 1 | Low |
| Mogessie et al. | 1 | 1 | 0 | 1 | 1 | 1 | 1 | 0 | 1 | Low |
| Mok et al. | 0 | 1 | 0 | 0 | 1 | 1 | 1 | 0 | 1 | Moderate |
| Momtaz et al. | 0 | 1 | 0 | 0 | 1 | 1 | 1 | 0 | 1 | Moderate |
| Moorkerjee et al. | 0 | 1 | 0 | 0 | 1 | 1 | 1 | 0 | 1 | Moderate |
| Moorkerjee et al. 2 | 0 | 1 | 0 | 0 | 1 | 1 | 1 | 0 | 1 | Moderate |
| Nayak et al. | 0 | 1 | 0 | 0 | 1 | 1 | 1 | 1 | 1 | Moderate |
| Ng et al. | 0 | 1 | 0 | 0 | 1 | 1 | 1 | 0 | 1 | Moderate |
| Onyuka et al. | 0 | 1 | 1 | 0 | 1 | 1 | 1 | 0 | 1 | Moderate |
| Pal et al. 2 | 0 | 1 | 0 | 0 | 1 | 1 | 1 | 1 | 1 | Moderate |
| Palit et al. | 0 | 1 | 1 | 0 | 1 | 1 | 1 | 1 | 1 | Low |
| Potgieter et al. | 0 | 1 | 0 | 0 | 1 | 1 | 1 | 1 | 1 | Moderate |
| Rafique et al. | 1 | 1 | 0 | 0 | 1 | 1 | 1 | 1 | 1 | Low |
| Rai et al. | 1 | 1 | 0 | 0 | 1 | 1 | 1 | 0 | 1 | Moderate |
| Rasheed et al. | 0 | 1 | 0 | 0 | 1 | 1 | 1 | 0 | 0 | Moderate |
| Sacheli et al. | 0 | 1 | 0 | 0 | 1 | 1 | 1 | 0 | 1 | Moderate |
| Saima et al. | 1 | 1 | 0 | 0 | 1 | 1 | 1 | 1 | 1 | Low |
| Saravanan et al. | 0 | 1 | 0 | 0 | 1 | 1 | 1 | 0 | 1 | Moderate |
| Schriewer et al. | 0 | 1 | 0 | 0 | 1 | 1 | 1 | 1 | 1 | Moderate |
| Shanan et al. | 0 | 1 | 0 | 0 | 1 | 1 | 1 | 0 | 1 | Moderate |
| Shishir et al. | 0 | 1 | 0 | 0 | 1 | 1 | 1 | 0 | 1 | Moderate |
| Singh and Lin | 0 | 1 | 1 | 0 | 1 | 1 | 1 | 1 | 0 | Moderate |
| Sirajul et al. | 0 | 1 | 0 | 0 | 1 | 1 | 1 | 0 | 1 | Moderate |
| Sorensen et al. | 0 | 1 | 0 | 0 | 1 | 1 | 1 | 0 | 1 | Moderate |
| Ssemanda et al. | 0 | 1 | 0 | 0 | 1 | 1 | 1 | 0 | 1 | Moderate |
| Taviani et al. | 0 | 1 | 0 | 0 | 1 | 1 | 1 | 0 | 0 | Moderate |
| Teklehaimanot et al. | 0 | 1 | 0 | 0 | 1 | 1 | 1 | 0 | 1 | Moderate |
| Thongchankaew et al. | 0 | 1 | 0 | 0 | 1 | 1 | 1 | 1 | 1 | Moderate |
| Torresi et al. | 0 | 1 | 0 | 0 | 1 | 1 | 1 | 1 | 1 | Moderate |
| Wang et al. | 0 | 1 | 0 | 0 | 1 | 1 | 1 | 1 | 1 | Moderate |
| Waturangi et al. | 0 | 1 | 0 | 0 | 1 | 1 | 1 | 1 | 1 | Moderate |
| Whitehouse et al. | 0 | 1 | 0 | 0 | 1 | 1 | 1 | 1 | 1 | Moderate |
| Wongworapat et al. | 0 | 1 | 0 | 0 | 1 | 1 | 1 | 0 | 1 | Moderate |
| Wu et al. | 0 | 1 | 0 | 0 | 1 | 1 | 1 | 0 | 1 | Moderate |
| Yan et al. | 0 | 1 | 0 | 0 | 1 | 1 | 1 | 0 | 0 | Moderate |
| Yue et al. | 1 | 1 | 0 | 0 | 1 | 1 | 1 | 1 | 1 | Low |

**References**

1. Abana D, Gyamfi E, Dogbe M, et al. Investigating the virulence genes and antibiotic susceptibility patterns of Vibrio cholerae O1 in environmental and clinical isolates in Accra, Ghana. *BMC Infectious Diseases*; 19. Epub ahead of print 2019. DOI: 10.1186/s12879-019-3714-z.

2. Ahmad B, Liaquat M, Ali J, et al. Microbiology and evaluation of antibiotic resistant bacterial profiles of drinking water in Peshawar, Khyber Pakhtunkhwa. *World Applied Sciences Journal* 2014; 30: 1668–1677.

3. Ahmed J, Wong LP, Chua YP, et al. Quantitative Microbial Risk Assessment of Drinking Water Quality to Predict the Risk of Waterborne Diseases in Primary-School Children. *Int J Environ Res Public Health*; 17. Epub ahead of print 17 April 2020. DOI: 10.3390/ijerph17082774.

4. Akoachere J-FTK, Mbuntcha CKP. Water sources as reservoirs of Vibrio cholerae O1 and non-O1 strains in Bepanda, Douala (Cameroon): relationship between isolation and physico-chemical factors. *BMC Infect Dis* 2014; 14: 421.

5. Alam MT, Weppelmann TA, Longini I, et al. Increased isolation frequency of toxigenic Vibrio cholerae O1 from environmental monitoring sites in Haiti. *PLoS One* 2015; 10: e0124098.

6. Alaoui HL, Oufdou K, Mezrioui N-E. Determination of several potential virulence factors in non-o1 Vibrio cholerae, Pseudomonas aeruginosa, faecal coliforms and streptococci isolated from Marrakesh groundwater. *Water Science and Technology* 2010; 61: 1895–1905.

7. Aulet O, Silva C, Fraga SG, et al. Detection of viable and viable nonculturable Vibrio cholerae O1 through cultures and immunofluorescence in the Tucumán rivers, Argentina. *Rev Soc Bras Med Trop* 2007; 40: 385–390.

8. Bahk YY, Kim HS, Rhee O-J, et al. Long-Term Monitoring of Noxious Bacteria for Construction of Assurance Management System of Water Resources in Natural Status of the Republic of Korea. *J Microbiol Biotechnol* 2020; 30: 1516–1524.

9. Bauza V, Madadi V, Ocharo R, et al. Enteric pathogens from water, hands, surface, soil, drainage ditch, and stream exposure points in a low-income neighborhood of Nairobi, Kenya. *SCIENCE OF THE TOTAL ENVIRONMENT*; 709. Epub ahead of print 20 March 2020. DOI: 10.1016/j.scitotenv.2019.135344.

10. Bhandari M, Rathnayake IU, Ariotti L, et al. Toxigenic Vibrio cholerae strains in South-East Queensland, Australian river waterways. *Appl Environ Microbiol* 2023; 89: e0047223.

11. Bisimwa AM, Kisuya B, Kazadi ZM, et al. Monitoring faecal contamination and relationship of physicochemical variables with faecal indicator bacteria numbers in Bukavu surface waters, tributaries of Lake Kivu in Democratic Republic of Congo. *Hygiene and Environmental Health Advances*; 3. Epub ahead of print 2022. DOI: 10.1016/j.heha.2022.100012.

12. Bliem R, Reischer G, Linke R, et al. Spatiotemporal dynamics of Vibrio cholerae in turbid alkaline lakes as determined by quantitative PCR. *Applied and Environmental Microbiology*; 84. Epub ahead of print 2018. DOI: 10.1128/AEM.00317-18.

13. Böer SI, Heinemeyer E-A, Luden K, et al. Temporal and Spatial Distribution Patterns of Potentially Pathogenic Vibrio spp. at Recreational Beaches of the German North Sea. *Microbial Ecology* 2013; 65: 1052–1067.

14. Bwire G, Debes AK, Orach CG, et al. Environmental Surveillance of Vibrio cholerae O1/O139 in the Five African Great Lakes and Other Major Surface Water Sources in Uganda. *Front Microbiol* 2018; 9: 1560.

15. Chandran A, Hatha AAM, Varghese S. Increased prevalence of indicator and pathogenic bacteria in Vembanadu Lake: A function of salt water regulator, along south west coast of India. *Journal of Water and Health* 2008; 6: 539–546.

16. Chaturongkasumrit Y, Techaruvichit P, Takahashi H, et al. Microbiological evaluation of water during the 2011 flood crisis in Thailand. *Science of the Total Environment* 2013; 463–464: 959–967.

17. Chigbu LN, Iroegbu CU. Vibrio species from diarrhoeal stools and water environment in Cross River State, Nigeria. *International Journal of Environmental Health Research* 2000; 10: 219–228.

18. Chomvarin C, Namwat W, Wongwajana S, et al. Application of duplex-PCR in rapid and reliable detection of toxigenic vibrio cholerae in water samples in Thailand. *Journal of General and Applied Microbiology* 2007; 53: 229–237.

19. Coly I, Gassama Sow A, Seydi M, et al. Vibrio cholerae and vibrio parahaemolyticus detected in seafood products from Senegal. *Foodborne Pathogens and Disease* 2013; 10: 1050–1058.

20. Dickinson G, Lim K-Y, Jiang SC. Quantitative microbial risk assessment of pathogenic vibrios in marine recreational waters of southern california. *Appl Environ Microbiol* 2013; 79: 294–302.

21. du Preez M, van Der Merwe MR, Cumbana A, et al. A survey of Vibrio cholerae O1 and O139 in estuarine. *Water SA* 2010; 36: 615–620.

22. Dumontet S, Krovacek K, Svenson SB, et al. Prevalence and diversity of Aeromonas and Vibrio spp. in coastal waters of Southern Italy. *Comparative Immunology, Microbiology and Infectious Diseases* 2000; 23: 53–72.

23. El-Sayed AKA, Abou-Dobara MI, Abdel-Malak CA, et al. Taqman hydrolysis probe application for Escherichia coli, salmonella enterica, and vibrio cholerae detection in surface and drinking water. *Journal of Water Sanitation and Hygiene for Development* 2019; 9: 492–499.

24. Fang L, Ginn AM, Harper J, et al. Survey and genetic characterization of Vibrio cholerae in Apalachicola Bay, Florida (2012–2014). *Journal of Applied Microbiology* 2019; 126: 1265–1277.

25. Faouzi J, Bedoui I, Rezouki S, et al. Vertical Transfer of Bacteriological and Parasitological Pollutants from Irrigation Water to Soil and Crops. *Ecological Engineering and Environmental Technology* 2023; 24: 93–103.

26. Ferdous J, Sultana R, Rashid RB, et al. A Comparative Analysis of Vibrio cholerae Contamination in Point-of-Drinking and Source Water in a Low-Income Urban Community, Bangladesh. *Front Microbiol* 2018; 9: 489.

27. Ferguson AS, Layton AC, Mailloux BJ, et al. Comparison of fecal indicators with pathogenic bacteria and rotavirus in groundwater. *Sci Total Environ* 2012; 431: 314–322.

28. Fernández-Delgado M, Suárez P, Giner S, et al. Occurrence and virulence properties of Vibrio and Salinivibrio isolates from tropical lagoons of the southern Caribbean Sea. *Antonie van Leeuwenhoek, International Journal of General and Molecular Microbiology* 2017; 110: 833–841.

29. Fraga SG, Pichel M, Costagliola M, et al. Environment and virulence factors of Vibrio cholerae strains isolated in Argentina. *J Appl Microbiol* 2007; 103: 2448–2456.

30. George CM, Hasan K, Monira S, et al. A prospective cohort study comparing household contact and water Vibrio cholerae isolates in households of cholera patients in rural Bangladesh. *PLoS Neglected Tropical Diseases*; 12. Epub ahead of print 2018. DOI: 10.1371/journal.pntd.0006641.

31. Gil AI, Louis VR, Rivera ING, et al. Occurrence and distribution of Vibrio cholerae in the coastal environment of Peru. *Environmental Microbiology* 2004; 6: 699–706.

32. Goh SG, Bayen S, Burger D, et al. Occurrence and distribution of bacteria indicators, chemical tracers and pathogenic vibrios in Singapore coastal waters. *Marine Pollution Bulletin* 2017; 114: 627–634.

33. Grothen DC, Zach SJ, Davis PH. Detection of Intestinal Pathogens in River, Shore, and Drinking Water in Lima, Peru. *J Genomics* 2017; 5: 4–11.

34. Halder M, Mookerjee S, Batabyal P, et al. Environmental Vibrio cholerae non O1/ non O139 from the Gangetic delta: a diarrhoeal disease purview. *International Journal of Environmental Health Research* 2017; 27: 241–251.

35. Hosen MA, Ovi FK, Rashid H, et al. Characterization of Vibrio spp. in environmental water samples collected from flood prone areas of Bangladesh and their antibiotic resistance profile. *AIMS Microbiol* 2021; 7: 471–480.

36. Hounmanou YMG, Leekitcharoenphon P, Hendriksen RS, et al. Surveillance and Genomics of Toxigenic Vibrio cholerae O1 From Fish, Phytoplankton and Water in Lake Victoria, Tanzania. *Front Microbiol* 2019; 10: 901.

37. Islam M, Sakakibara H, Karim M, et al. Bacteriological assessment of drinking water supply options in coastal areas of Bangladesh. *JOURNAL OF WATER AND HEALTH* 2011; 9: 415–428.

38. Jesudason MV, Balaji V, Mukundan U, et al. Ecological study of Vibrio cholerae in Vellore. *Epidemiol Infect* 2000; 124: 201–206.

39. Kaboré S, Cecchi P, Mosser T, et al. Occurrence of Vibrio cholerae in water reservoirs of Burkina Faso. *Res Microbiol* 2018; 169: 1–10.

40. Kachienga L, Prosperit M, Traore A, et al. Assessment of the presence of Vibrio cholera and detection of toxigenic Vibrio cholerae in river sources within the Vhembe District Municipality Limpopo province of South Africa. *JOURNAL OF WATER AND HEALTH* 2024; 22: 1248–1260.

41. Kaddumukasa M, Nsubuga D, Muyodi FJ. Occurence of Culturable Vibrio cholerae from Lake Victoria, and Rift Valley Lakes Albert and George, Uganda. *Lakes and Reservoirs: Science, Policy and Management for Sustainable Use* 2012; 17: 291–299.

42. Kahler AM, Haley BJ, Chen A, et al. Environmental surveillance for toxigenic Vibrio cholerae in surface waters of Haiti. *Am J Trop Med Hyg* 2015; 92: 118–125.

43. Keawvichit R, Wongworapat K, Putsyainant P, et al. Parasitic and bacterial contamination in collards using effluent from treated domestic wastewater in Chiang Mai, Thailand. *Southeast Asian Journal of Tropical Medicine and Public Health* 2001; 32: 240–244.

44. Kim N-Y, Shi HJ, Oh S-S, et al. Wastewater Knows Pathogen Spread: Analysis of Residential Wastewater for Infectious Microorganisms including SARS-CoV-2. *Infection and Chemotherapy* 2023; 55: 214–225.

45. Kirschner A, Pleininger S, Jakwerth S, et al. Application of three different methods to determine the prevalence, the abundance and the environmental drivers of culturable Vibrio cholerae in fresh and brackish bathing waters. *JOURNAL OF APPLIED MICROBIOLOGY* 2018; 125: 1186–1198.

46. Kokashvili T, Whitehouse CA, Tskhvediani A, et al. Occurrence and Diversity of Clinically Important Vibrio Species in the Aquatic Environment of Georgia. *Frontiers in Public Health*; 3. Epub ahead of print 2015. DOI: 10.3389/fpubh.2015.00232.

47. Lee SH, Lee HJ, Myung GE, et al. Distribution of Pathogenic Vibrio Species in the Coastal Seawater of South Korea (2017-2018). *Osong Public Health Res Perspect* 2019; 10: 337–342.

48. Lipp EK, Rivera ING, Gil AI, et al. Direct detection of Vibrio cholerae and ctxA in Peruvian coastal water and plankton by PCR. *Applied and Environmental Microbiology* 2003; 69: 3676–3680.

49. Luo Y, Wang H, Liang J, et al. Population Structure and Multidrug Resistance of Non-O1/Non-O139 Vibrio cholerae in Freshwater Rivers in Zhejiang, China. *Microbial Ecology* 2021; 82: 319–333.

50. Malayil L, Turner JW, Mote BL, et al. Evaluation of enrichment media for improved PCR-based detection of V. cholerae and V. vulnificus from estuarine water and plankton. *J Appl Microbiol* 2011; 110: 1470–1475.

51. Mathews EB, Sunil B, Prejit N, et al. Occurrence and Antibiotic Susceptibility Testing of Vibrio cholerae from District Wayanad, Kerala, India. *Proceedings of the National Academy of Sciences India Section B - Biological Sciences* 2018; 88: 673–678.

52. Mishra A, Taneja N, Sharma M. Demonstration of viable but nonculturable Vibrio cholerae O1 in fresh water environment of India using ciprofloxacin DFA-DVC method. *Letters in Applied Microbiology* 2011; 53: 124–126.

53. Mogessie H, Legesse M, Hailu AF, et al. Vibrio cholerae O1 and Escherichia coli O157:H7 from drinking water and wastewater in Addis Ababa, Ethiopia. *BMC Microbiol* 2024; 24: 219.

54. Mok JS, Ryu A, Kwon JY, et al. Distribution of Vibrio species isolated from bivalves and bivalve culture environments along the Gyeongnam coast in Korea: Virulence and antimicrobial resistance of Vibrio parahaemolyticus isolates. *Food Control*; 106. Epub ahead of print 2019. DOI: 10.1016/j.foodcont.2019.06.023.

55. Momtaz H, Dehkordi FS, Rahimi E, et al. Detection of Escherichia coli, Salmonella species, and Vibrio cholerae in tap water and bottled drinking water in Isfahan, Iran. *BMC Public Health*; 13. Epub ahead of print 2013. DOI: 10.1186/1471-2458-13-556.

56. Mookerjee S, Batabyal P, Sarkar MH, et al. Seasonal prevalence of enteropathogenic Vibrio and their phages in the riverine estuarine ecosystem of south Bengal. *PLoS ONE*; 10. Epub ahead of print 2015. DOI: 10.1371/journal.pone.0137338.

57. Mookerjee S, Jaiswal A, Batabyal P, et al. Seasonal dynamics of Vibrio cholerae and its phages in riverine ecosystem of Gangetic West Bengal: cholera paradigm. *Environmental Monitoring and Assessment* 2014; 186: 6241–6250.

58. Nayak SR, Nayak AK, Biswal BL, et al. Incidence of Bacterial Enteropathogens among Diarrhea Patients from Tribal Areas of Odisha. *Jpn J Infect Dis* 2020; 73: 263–267.

59. Ng C, Goh SG, Saeidi N, et al. Occurrence of Vibrio species, beta-lactam resistant Vibrio species, and indicator bacteria in ballast and port waters of a tropical harbor. *Science of the Total Environment* 2018; 610–611: 651–656.

60. Onyuka JHO, Kakai R, Onyango DM, et al. Prevalence and antimicrobial susceptibility patterns of enteric bacteria isolated from water and fish in lake victoria basin of western kenya. *World Academy of Science, Engineering and Technology* 2011; 51: 761–768.

61. Pal BB, Nayak SR, Biswal B, et al. Environmental reservoirs of Vibrio cholerae serogroups in the flowing freshwater environs from the tribal areas of Odisha, Eastern India. *Environmental Microbiology Reports* 2021; 13: 119–125.

62. Palit A, Batabyal P, Kanungo S, et al. In-house contamination of potable water in urban slum of Kolkata, India: A possible transmission route of diarrhea. *Water Science and Technology* 2012; 66: 299–303.

63. Potgieter N, Karambwe S, Mudau L, et al. Human Enteric Pathogens in Eight Rivers Used as Rural Household Drinking Water Sources in the Northern Region of South Africa. *INTERNATIONAL JOURNAL OF ENVIRONMENTAL RESEARCH AND PUBLIC HEALTH*; 17. Epub ahead of print 2020. DOI: 10.3390/ijerph17062079.

64. Rafique R, Rashid M, Monira S, et al. Transmission of infectious vibrio cholerae through drinking water among the household contacts of cholera patients (CHoBI7 Trial). *Frontiers in Microbiology*; 7. Epub ahead of print 2016. DOI: 10.3389/fmicb.2016.01635.

65. Rai KR, Mukhiya RK, Thapa S, et al. Diarrheal disease outbreak in Gaidatar village of Rautahat District, Nepal. *BMC Research Notes*; 12. Epub ahead of print 2019. DOI: 10.1186/s13104-019-4156-9.

66. Rasheed F, Khan A, Kazmi S. Bacteriological analysis, antimicrobial susceptibility and detection of 16S rRNA gene of Helicobacter pylori by PCR in drinking water samples of earthquake affected areas and other parts of Pakistan. *MALAYSIAN JOURNAL OF MICROBIOLOGY* 2009; 5: 123–127.

67. Sacheli R, Philippe C, Meex C, et al. Occurrence of Vibrio spp. in Selected Recreational Water Bodies in Belgium during 2021 Bathing Season. *International Journal of Environmental Research and Public Health*; 20. Epub ahead of print 2023. DOI: 10.3390/ijerph20206932.

68. Saima S, Ferdous J, Sultana R, et al. Detecting Enteric Pathogens in Low-Risk Drinking Water in Dhaka, Bangladesh: An Assessment of the WHO Water Safety Categories. *Tropical Medicine and Infectious Disease*; 8. Epub ahead of print 2023. DOI: 10.3390/tropicalmed8060321.

69. Saravanan V, Sanath Kumar H, Karunasagar I, et al. Putative virulence genes of Vibrio cholerae from seafoods and the coastal environment of Southwest India. *International Journal of Food Microbiology* 2007; 119: 329–333.

70. Schriewer A, Miller WA, Byrne BA, et al. Presence of Bacteroidales as a predictor of pathogens in surface waters of the central California coast. *Applied and Environmental Microbiology* 2010; 76: 5802–5814.

71. Shanan S, Abd H, Hedenström I, et al. Detection of Vibrio cholerae and Acanthamoeba species from same natural water samples collected from different cholera endemic areas in Sudan. *BMC Research Notes*; 4. Epub ahead of print 2011. DOI: 10.1186/1756-0500-4-109.

72. Shishir MA, Mamun MA, Mian MM, et al. Prevalence of Vibrio cholerae in Coastal Alternative Supplies of Drinking Water and Association with Bacillus-Like Spore Formers. *Frontiers in Public Health*; 6. Epub ahead of print 2018. DOI: 10.3389/fpubh.2018.00050.

73. Singh A, Lin J. Microbiological, coliphages and physico-chemical assessments of the Umgeni River, South Africa. *International Journal of Environmental Health Research* 2015; 25: 33–51.

74. Sirajul Islam M, Brooks A, Kabir MS, et al. Faecal contamination of drinking water sources of Dhaka city during the 2004 flood in Bangladesh and use of disinfectants for water treatment. *Journal of Applied Microbiology* 2007; 103: 80–87.

75. Sorensen JPR, Lapworth DJ, Read DS, et al. Tracing enteric pathogen contamination in sub-Saharan African groundwater. *Science of the Total Environment* 2015; 538: 888–895.

76. Ssemanda JN, Reij MW, van Middendorp G, et al. Foodborne pathogens and their risk exposure factors associated with farm vegetables in Rwanda. *Food Control* 2018; 89: 86–96.

77. Taviani E, van den Berg H, Nhassengo F, et al. Occurrence of waterborne pathogens and antibiotic resistance in water supply systems in a small town in Mozambique. *BMC MICROBIOLOGY*; 22. Epub ahead of print 2022. DOI: 10.1186/s12866-022-02654-3.

78. Teklehaimanot GZ, Genthe B, Kamika I, et al. Prevalence of enteropathogenic bacteria in treated effluents and receiving water bodies and their potential health risks. *Science of the Total Environment* 2015; 518: 441–449.

79. Thongchankaew U, Mittraparp-Arthorn P, Sukhumungoon P, et al. Occurrence of potentially pathogenic vibrios and related environmental factors in Songkhla Lake, Thailand. *Canadian Journal of Microbiology* 2011; 57: 867–873.

80. Torresi M, Sperandii A, Ricci L, et al. Detection and characterisation of potentially pathogenic species of vibrio in the vibrata river, Abruzzo region, Italy. *Veterinaria Italiana* 2018; 54: 125–135.

81. Wang Q, Fu S, Yang Q, et al. The impact of water intrusion on pathogenic vibrio species to inland brackish waters of China. *International Journal of Environmental Research and Public Health* 2020; 17: 1–14.

82. Waturangi D, Pradita N, Linarta J, et al. Prevalence and Molecular Characterization of Vibrio cholerae from Ice and Beverages Sold in Jakarta, Indonesia, Using Most Probable Number and Multiplex PCR. *JOURNAL OF FOOD PROTECTION* 2012; 75: 651–659.

83. Whitehouse CA, Baldwin C, Sampath R, et al. Identification of pathogenic Vibrio species by multilocus PCR-electrospray ionization mass spectrometry and its application to aquatic environments of the former Soviet Republic of Georgia. *Applied and Environmental Microbiology* 2010; 76: 1996–2001.

84. Wongworapat K, Keawvichit R, Putsyanant P, et al. Examination for intestinal parasites and enteric bacteria in the wastewater and treated wastewater from the city of Chiang Mai, Thailand. *Southeast Asian Journal of Tropical Medicine and Public Health* 2001; 32: 236–239.

85. Wu G, Yang J, Jiang H, et al. Distribution of potentially pathogenic bacteria in the groundwater of the Jianghan Plain, central China. *International Biodeterioration and Biodegradation*; 143. Epub ahead of print 2019. DOI: 10.1016/j.ibiod.2019.05.028.

86. Yan L, Pei X, Zhang X, et al. Occurrence of four pathogenic Vibrios in Chinese freshwater fish farms in 2016. *Food Control* 2019; 95: 85–89.

87. Yue Y, Gong J, Wang D, et al. Influence of climate factors on Vibrio cholerae dynamics in the Pearl River estuary, South China. *World Journal of Microbiology and Biotechnology* 2014; 30: 1797–1808.
